# Irisin is a pro-myogenic factor that induces skeletal muscle hypertrophy and rescues denervation-induced atrophy

**DOI:** 10.1038/s41467-017-01131-0

**Published:** 2017-10-24

**Authors:** Musarrat Maisha Reza, Nathiya Subramaniyam, Chu Ming Sim, Xiaojia Ge, Durgalakshmi Sathiakumar, Craig McFarlane, Mridula Sharma, Ravi Kambadur

**Affiliations:** 1 School of Biological Sciences, Nanyang Technological University, 60 Nanyang Drive, Singapore, 637551 Singapore; 2 Singapore Institute for Clinical Sciences (A*STAR), Brenner Centre for Molecular Medicine, 30 Medical Drive, Singapore, 117609 Singapore; 3 Department of Biochemistry, YLL School of Medicine, National University of Singapore, 8 Medical Drive, Singapore, 117596 Singapore; 4 Present Address: Department of Molecular & Cell Biology, College of Public Health, Medical and Veterinary Sciences, James Cook University, Townsville, 4811 QLD Australia

## Abstract

Exercise induces expression of the myokine irisin, which is known to promote browning of white adipose tissue and has been shown to mediate beneficial effects following exercise. Here we show that irisin induces expression of a number of pro-myogenic and exercise response genes in myotubes. Irisin increases myogenic differentiation and myoblast fusion via activation of IL6 signaling. Injection of irisin in mice induces significant hypertrophy and enhances grip strength of uninjured muscle. Following skeletal muscle injury, irisin injection improves regeneration and induces hypertrophy. The effects of irisin on hypertrophy are due to activation of satellite cells and enhanced protein synthesis. In addition, irisin injection rescues loss of skeletal muscle mass following denervation by enhancing satellite cell activation and reducing protein degradation. These data suggest that irisin functions as a pro-myogenic factor in mice.

## Abstract (web summary)

Exercise induces expression of the myokine Irisin. Here the authors show that Irisin promotes muscle hypertrophy and regeneration following injury or denervation in mice, by activating satellite cells and modulating protein synthesis and degradation.

## Introduction

Skeletal muscle is one of the largest organs in the human body. Skeletal muscle is made up of multi-nucleated muscle fibers, which are formed by the fusion of myoblasts during embryonic and fetal development^1^. During postnatal myogenesis, muscle stem cells, also known as satellite cells are activated upon myo-trauma, exercise, or stress to form myoblasts, which differentiate and eventually give rise to new muscle fibers^2^. Exercise imparts many health benefits to skeletal muscle. One of the hallmarks of exercise is induction of hypertrophy of skeletal muscle. During exercise, muscle fibers undergo necrosis, which activates satellite cells in order to repair the damaged fibers^3^. Hypertrophy of skeletal muscle can be attributed to increased protein synthesis via the Akt/mTOR pathway^4^ and also the Erk1/2 pathway^5^. Increased phosphorylation of molecules in these pathways leads to the activation of signal transduction pathways that increase protein synthesis. Additionally, protein degradation is inhibited during exercise^6, 7^. Increased protein synthesis coupled with reduced protein degradation are significant contributing factors to skeletal muscle hypertrophy^8^, which is consistent with the exercise phenotype.

Exercise induces the expression of the nuclear transcriptional co-activator peroxisome proliferator-activated receptor-γ co-activator-1-α (PGC1-α) in skeletal muscle fibers^9^. A recent study revealed that PGC1-α induces the expression of fibronectin type III domain containing 5 (*Fndc5*), which encodes for a 209-amino-acid (aa)-long, type I membrane protein FNDC5^10^. Boström et al. further showed that FNDC5 is cleaved at the C terminus to give rise to a 112-aa-long secreted hormone known as irisin. Irisin induces the expression of “browning genes”, such as *Ucp1*, *Cidea*, *Cpt1b*, and *Dio2*, in white adipocytes, which is an effect partly mediated by peroxisome proliferator-activated receptor-α^10^. Upregulation of Ucp1 promotes uncoupling of oxidative phosphorylation from energy production, resulting in release of energy as heat via non-shivering thermogenesis and enhanced browning of white adipose tissue^9, 11, 12^. Consistent with this result, secretion of irisin from skeletal muscle during exercise has been recently shown to have evolved from shivering thermogenesis, where muscle contracts in order to generate heat when exposed to the cold^13^. Although initially identified as a myokine, recently it has been shown that small amounts of irisin are synthesized and secreted from adipose tissue^14^ and liver^15^.

While the presence of irisin and increased expression of irisin in response to exercise has been well established in mouse models, contrasting results have been reported in humans. Boström et al.^10^ noted the presence of irisin in human serum and increased serum irisin levels in humans after exercise. Rachke et al. observed that *FNDC5* sequence is found in most rodents and primates, and is conserved throughout the species. However, in human *FNDC5* sequence, the translational start codon ATG is not conserved and instead ATA, which codes for isoleucine^16^, is found. Rachke et al. also reported that upregulation of PGC1-α mRNA expression by simulating contraction of primary human myotubes did not show a concomitant increase in the FNDC5 mRNA expression. Furthermore, Rachke et al. failed to observe an increase in *FNDC5* mRNA in either endurance or strength training (resistance exercise). Hence, they concluded that irisin may not confer its beneficial effects in humans^16^. Albrecht et al. also cast doubts on the presence of circulating irisin since all previous studies used commercial enzyme-linked immunosorbent assay (ELISA) kits using polyclonal antibodies that were not tested previously for irisin specificity in serum and they could very well display cross reactivity^17^.

Jedrychowski et al. then responded very elegantly to these observations by using tandem mass spectrometry to detect irisin with control peptides enriched with heavy stable isotopes^18^. Irisin was detected at 12 kDa and it was reported that irisin circulates in the human serum at about 3.6 ng/ml in sedentary individuals and is increased after aerobic interval training to 4.3 ng/ml. To date 255 publications (Pubmed search) confirm the presence of human irisin. Hence, it is possible that irisin may also play a crucial role in metabolism in humans^19^.

Irisin has been shown to be upregulated by both endurance and resistance exercise. Furthermore, circulating irisin levels have been positively correlated with biceps circumference and insulin-like growth factor-1 levels in humans^15^. Similarly, in *myostatin*-null (*Mstn*-null) mice with increased musculature, elevated irisin levels are seen^20^. In addition, recent work has revealed that irisin is able to stimulate muscle growth-related genes in humans^20^. It has been further shown that irisin levels are increased during myogenic differentiation and that irisin treatment results in increased p-Erk expression, which is involved in the protein synthesis pathway^21^. Taken together, these studies provide clues that increased irisin levels could promote skeletal muscle growth.

Since exercise induces FNDC5/irisin expression and promotes hypertrophy, we hypothesized that recombinant irisin could potentially induce skeletal muscle hypertrophy and as such will have therapeutic benefit in overcoming atrophy. In order to investigate this, we utilized recombinant murine irisin protein. Here we have shown that irisin promotes myogenic differentiation by improving myoblast fusion. Furthermore, injecting irisin induced skeletal muscle hypertrophy and enhanced skeletal muscle regeneration after muscle injury.

## Results

### Irisin treatment induces the expression of *Ucp1*

**Fig. 1:**
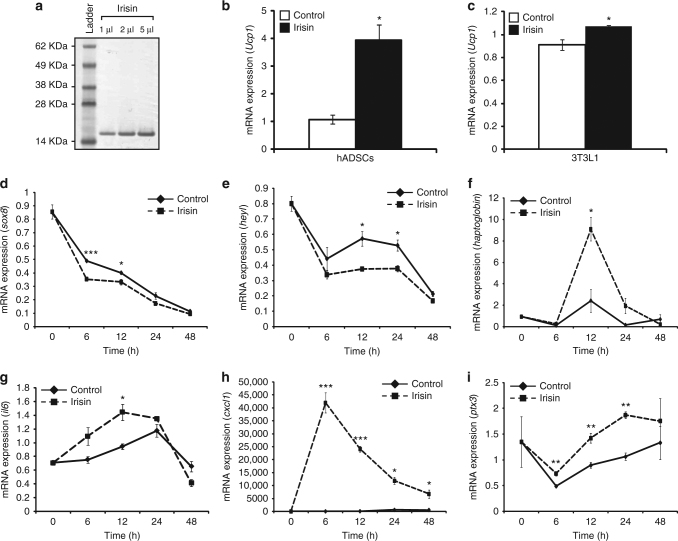
Irisin induces gene expression changes and improves myogenesis. **a** Image of Coomassie-stained polyacrylamide gel showing recombinant His-tagged irisin protein. Recombinant irisin protein was detected as a single band at ~15 kDa. Lane 1 shows the SeeBlue Plus 2 Pre-Stained ladder. Lanes 2, 3, and 4 show 1, 2, and 5 μl of the purified His-tagged irisin protein, respectively. **b** Graph representing qPCR analysis of *Ucp1* expression in human subcutaneous white adipose-derived stem cells (hADSCs) after 21 days of adipogenic differentiation in the presence of DB (control) or irisin (*n* = 2 biological replicates). **c** Graph representing qPCR analysis of *Ucp1* in 3T3L1 fibroblasts after 4 days of adipogenic differentiation in the presence of DB (control) or irisin (*n* = 3 biological replicates). Graphs displaying qPCR analysis of *sox8* (**d**), *heyL* (**e**), *haptoglobin* (**f**), *il6* (**g**), *cxcl1* (**h**), and *ptx3* (**i**) in 72 h-differentiated C2C12 myotubes treated with DB (control) or recombinant irisin protein for 0, 6, 12, 24, and 48 h. All qPCR graphs show gene expression normalized to *gapdh* (*n* = 3 biological replicates). Error bars represent mean ± SEM. Student’s *t*-test was performed for **b** and all relevant figure panels between **d** and **i**, and one-way ANOVA was peformed for **c**. Significance is indicated with *p < 0.05, **p < 0.01 and ***p < 0.001

Polyacrylamide gel electrophoresis (PAGE) and Coomassie blue staining revealed a single purified irisin band running at ~ 15 kDa (Fig. 1a). At 1:100 dilution of the purified recombinant irisin protein, low endotoxin levels of 1 was found^22^. In order to test the biological activity of our recombinant murine irisin protein, we treated differentiated human adipose-derived stem cells (hADSCs) with recombinant irisin protein. A significant ~4-fold increase in *Ucp1* expression was noted in hADSCs after treatment with irisin (Fig. 1b). We further noted a significant ~ 18% increase in *Ucp1* expression in irisin-treated 3T3L1 fibroblasts during adipogenic differentiation (Fig. 1c).

### Microarray analysis

To identify gene expression changes induced by irisin, we performed microarray on RNA isolated from either dialysis buffer (DB)- or irisin-treated C2C12 myotubes. Only genes that were upregulated or downregulated by ≥ 1.5-fold were analyzed for each time point. Of these selected genes we noted that genes involved in exercise, satellite cell regulation, skeletal muscle regeneration, muscle growth, and myogenesis were differentially expressed between DB- and irisin-treated myotubes (Table 1). A number of target genes identified from the microarray were validated through quantitative real-time PCR (qPCR). Subsequent qPCR analysis confirmed reduced expression of negative regulators of myogenic differentiation, including *sox8* (Fig. 1d) and *heyL* (Fig. 1e), following treatment with irisin. Moreover, exercise-induced and -secreted factors, such as haptoglobin (Fig. 1f) and interleukin 6 (*il6*; Fig. 1g), which promote skeletal muscle hypertrophy, were significantly upregulated by irisin treatment. The expression of C*xcl1*, a chemokine highly expressed in skeletal muscle and secreted during exercise, was elevated at all time points analyzed, with a sharp significant increase in *Cxcl1* noted 6 h post irisin treatment (Fig. 1h). Another exercise-secreted factor, pentraxin-3 (*ptx3*) (Fig. 1i) also showed a marked upregulation at all irisin treatment time points.

**Table 1:** Irisin treatment promotes the expression of pro-myogenic and exercise-related genes in myotubes

Exercise regulated	Gene (symbol)	6 h	12 h	24 h	48 h
Upregulated	*Cxcl1*	142.7	153.0	76.8	93.1
	*Cd74*	2.6	2.7	3.5	3.0
	*Nrg1*	—	—	1.8	2.9
	*Nrp*	—	—	—	2.9
	*Thbd*	1.5	2.0	1.5	2.4
	*Gap43*	−1.6	—	—	2.3
	*Socs3*	—	2.1	3.1	2.2
	*Ccnd1*	2.4	—	—	−1.6
	*Tiparp*	—	—	2.9	—
	*Sod3*	2.3	2.4	1.7	—
	*Timp1*	2.1	1.6	—	1.5
	*Mmp10*	3.3	—	—	—
	*Hp*	—	1.8	3.4	—
	*Prdx6*	—	−1.5	3.3	—
	*Ptx3*	2.5	5.1	8.9	6.8
Downregulated	*Crispld2*	−1.8	—	−3.0	−3.5
	*Cobll1*	—	−1.6	−2.6	—
	*Irak4*	—	—	−1.6	—
	*Gpd1l*	—	−2.1	—	—
	*Pdgfb*	—	—	−1.6	−1.6
Satellite cell regulation	Gene (symbol)	6 h	12 h	24 h	48 h
Inhibit differentiation of satellite cells	*Heyl*	−2.9	−3.6	−3.1	−3.4
	*Ogn*	−2.3	−1.5	−1.9	−1.6
	*Sox8*	−1.5	−2.0	−2.6	−1.6
Skeletal muscle regeneration	Gene (symbol)	6 h	12 h	24 h	48 h
Upregulated	*C1s*	1.6	1.6	1.8	3.9
	*Ccl2*	38.5	40.7	13.2	3.9
	*Ccl7*	10.4	18.7	7.6	4.9
	*Cxcl16*	10.6	10.8	7.4	3.2
	*Mmp9*	22.6	10.6	5.2	—
	*Ereg*	3.5	—	—	—
	*Figf*	−1.6	2.6	2.2	1.9
	*Mt1*	2.9	2.3	1.9	1.7
	*Cxcl12*	—	2.8	4.3	2.5
	*Megf10*	−1.6	−1.8	−1.7	−2.0
Downregulated	*Plau*	−1.8	−2.0	−1.6	—
Muscle growth and myogenesis	Gene (symbol)	6 h	12 h	24 h	48 h
Hypertrophy and growth factors	*Sema3f*	1.8	—	1.5	1.6
	*Il6*	6.4	6.7	2.5	3.6
	*Il6ra*	1.6	—		
	*Shq1*	2.4	—	—	—
	*Il7*	2.0	1.5	1.7	2.4
	*Serpina3g*	17.8	7.1	8.4	7.9
	*Tgfbi*	—	—	1.7	2.4
	*Bmp4*	−4.3	1.9	2.6	2.1
	*Tnfrsf11b*	—	—	2.0	1.7
	*Agxt2*	—	2.6	3.4	—

Table listing differentially expressed genes, identified through microarray analysis, between myotubes treated with either DB (control) or irisin for 6, 12, 24, and 48 h. Genes have been manually separated into exercise regulated, satellite cell regulation, skeletal muscle regeneration, and muscle growth and myogenesis categories. Gene symbols and fold changes at each treatment time point are given. Negative numbers reflect fold repression of gene expression. Only genes that were upregulated or downregulated by ≥ 1.5-fold were selected

### Irisin enhances myoblast proliferation and fusion

**Fig. 2:**
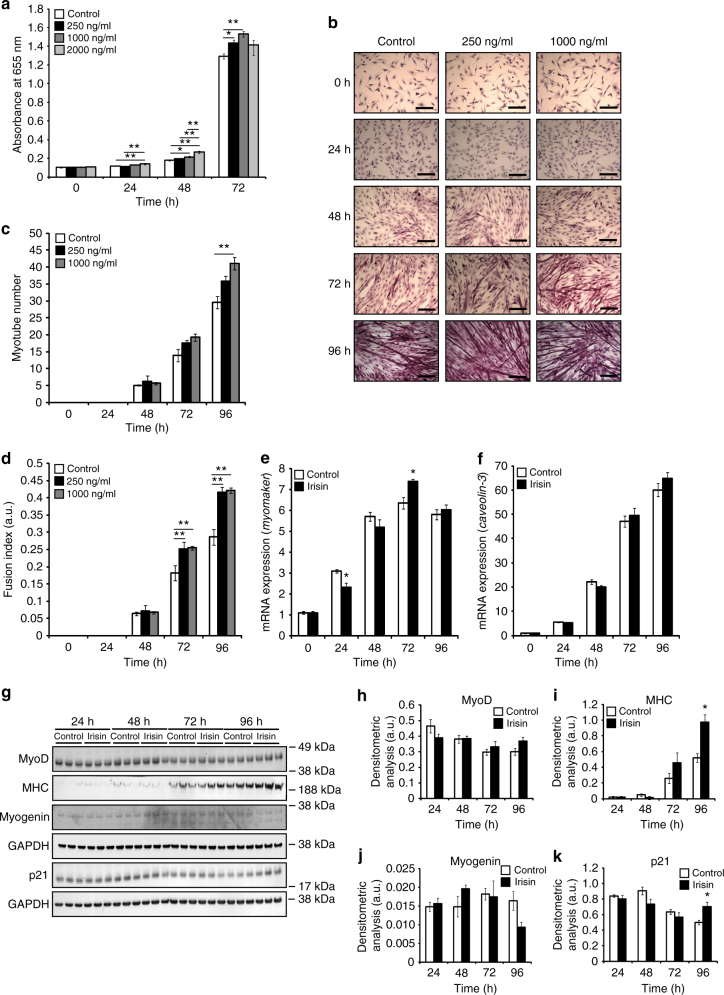
Irisin promotes skeletal muscle differentiation. **a** Graph showing the absorbance readings of cells treated with DB (control) or increasing concentrations of irisin (250, 1000, and 2000 ng/ml) at 0, 24, 48, and 72 h of proliferation. Absorbance reading at 655 nm is proportional to the number of cells present and hence, indicative of myoblast proliferation. (*n* = 16 biological replicates). **b** Representative images of H&E-stained myoblasts at 0, 24, 48, 72, and 96 h of differentiation in the presence of DB (control) or irisin (250 and 1000 ng/ml). **c** Graph showing quantification of myotube number at 0, 24, 48, 72, and 96 h of differentiation in the presence of DB (control) or irisin (250 and 1000 ng/ml) (*n* = 3 biological replicates). **d** Graph showing quantification of fusion index at 0, 24, 48, 72, and 96 h of differentiation in the presence of DB (control) or irisin (250 and 1000 ng/ml) (*n* = 3 biological replicates). Graphs displaying qPCR analysis of **e** *myomaker* and **f** *caveolin-3* expression normalized to GAPDH in differentiating myoblasts (0, 24, 48, 72, and 96 h) treated with DB (control) or irisin (1000 ng/ml) (*n* = 3 biological replicates). **g** IB analysis of MyoD, MHC, myogenin, and p21 protein levels. The levels of GAPDH were assessed as a loading control. Graphs show densitometric analysis of **h** MyoD, **i** MHC, **j** myogenin, and **k** p21 levels in arbitrary units (a.u), normalized to GAPDH (*n* = 3 biological replicates). Since MyoD, MHC, and myogenin IB analysis was performed on the same membrane, the same GAPDH was used to normalize the results. All IBs were performed on proteins collected from differentiating myotubes at 24, 48, 72, and 96 h treated with either DB (control) or irisin (1000 ng/ml). Error bars represent mean ± SEM. One-way ANOVA was performed for all relevant figure panels between **a** and **d**, and Student’s *t*-test was performed for all relevant figure panels between **e** and **k**. Significance is indicated with *p < 0.05 and **p < 0.01

The myoblast assay showed a significant increase in the number of myoblasts at 24, 48, and 72 h of proliferation when treated with irisin. Moreover, a dose-dependent increase in the number of myoblasts 48 h after treatment with increasing concentrations of irisin (250, 1000, and 2000 ng/ml) was observed when compared to DB-treated control (Fig. 2a). The optical density (OD) reading at 655 nm was proportional to the number of myoblasts in the different treatment groups.

To further investigate the effect of irisin on myogenesis, differentiating C2C12 myotubes were treated with irisin. A distinct increase in the number of myotubes was noted at both 72 and 96 h of differentiation in C2C12 myotubes treated with irisin, when compared to DB-treated controls (Fig. 2b, c). The increase in myotube number could be due to enhanced fusion. Consistent with this, a marked increase in myoblast fusion index was also observed at both 72 and 96 h of differentiation following irisin treatment (Fig. 2d). We further analyzed the gene expression of fusion markers. Irisin treatment resulted in significantly increased expression of the primary fusion marker, *myomaker* (Fig. 2e). Although we observed an increasing trend of the secondary fusion marker, *caveolin-3* (Fig. 2f), the increase was not statistically significant.

We next measured the levels of protein involved in myogenesis. MyoD, p21, myogenin, and myosin heavy chain (MHC) levels in proteins obtained from differentiating myotubes at 24, 48, 72, and 96 h treated with DB (control) or irisin were measured (Fig. 2g). We observed no distinct change in the expression of the myogenic markers, MyoD (Fig. 2h) and myogenin (Fig. 2j) after treatment with irisin. However, we observed a significant increase in p21 expression at 96 h (Fig. 2k). We noted an increase in the expression of MHC from 72 h, which became statistically significant at 96 h (Fig. 2i).

**Fig. 3:**
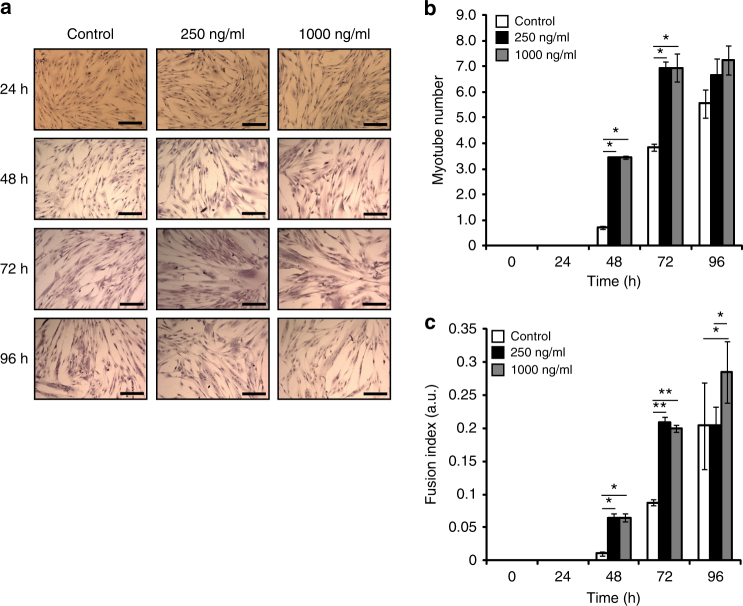
Irisin enhances myogenesis in primary human myoblast cultures. **a** Representative images of H&E-stained 36C15Q primary myoblasts at 24, 48, 72, and 96 h of differentiation in the presence of DB (control) or irisin (250 and 1000 ng/ml). **b** Graph showing quantification of myotube number at 0, 24, 48, 72, and 96 h of differentiation in the presence of DB (control) or irisin (250 and 1000 ng/ml). **c** Graph showing quantification of fusion index at 0, 24, 48, 72, and 96 h of differentiation in the presence of DB (control) or irisin (250 and 1000 ng/ml) (*n* = 2 biological replicates). Error bars represent mean ± SEM. One-way ANOVA was performed for all relevant figure panels. Significance is indicated with *p < 0.05 and **p < 0.01

To understand if recombinant murine irisin protein would result in a similar phenotype in humans, we treated differentiating 36C15Q primary human myotubes with DB or increasing concentrations of irisin (250 and 1000 ng/ml) (Fig. 3a). A significant increase in the myotube number at 48 and 72 h was observed (Fig. 3b). Fusion index of differentiating myotubes was also enhanced significantly at 48, 72, and 96 h (Fig. 3c). Our results suggest that recombinant irisin enhances myogenesis in both mouse and human myotubes.

### Irisin injection induces muscle hypertrophy

**Fig. 4:**
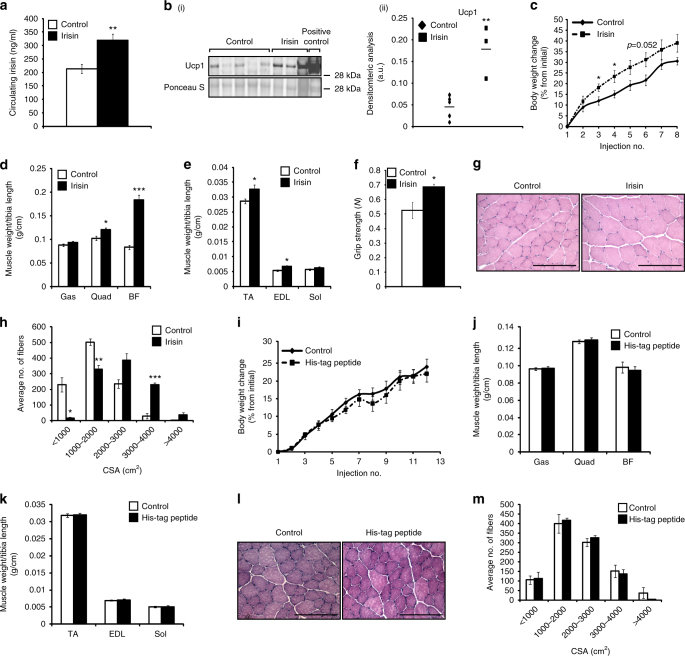
Irisin improves protein synthesis and reduces protein degradation. **a** Graph showing levels of irisin circulating in serum of mice injected with DB (control) or recombinant irisin (*n* = 5 mice for DB-injected group and *n* = 6 mice for irisin-injected group). **b** (i) IB analysis of Ucp1 levels. The levels of Ponceau S were assessed as a loading control. **b** (ii) Graph showing densitometric analysis of Ucp1 levels in arbitrary units (a.u), normalized to Ponceau S. Brown adipose tissue was used as a positive control to detect Ucp1 (*n* = 5 mice for DB-injected group and *n* = 3 mice for irisin-injected group). **c** Graph showing body weight change (% from initial) in mice injected with either DB or irisin protein. Graphs displaying **d** Gas, Quad, and BF, and **e** TA, EDL, and Sol muscle weights of mice injected with DB (control) or irisin. All hindlimb muscle weights were normalized to tibia length. **f** Graph showing the grip strength of mice (in newton; N) injected with DB (control) or irisin (*n* = 5 mice for DB-injected group and *n* = 4 mice for irisin-injected group). **g** Representative images of H&E-stained TA muscle from mice injected with DB (control) or irisin. Images were captured using a ×20 objective. Scale bar represents 100 μm. **h** Graph showing the distribution of TA myofiber CSA in mice injected with DB (control) or irisin (*n* = 3 mice per group). **i** Graph showing body weight change (% from initial) in mice injected with either DB (control) or His-tagged peptide. Graphs displaying **j** Gas, Quad, and BF, and **k** TA, EDL, and Sol muscle weights of mice injected with DB (control) or His-tagged peptide. All hindlimb muscle weights were normalized to tibia length (*n* = 5 mice for each treatment group). **l** Representative images of H&E-stained TA muscle from mice injected with DB (control) or His-tagged peptide. Images were captured using a ×20 objective. Scale bar represents 100 μm. **m** Graph showing the distribution of TA myofiber CSA in mice injected with DB (control) or His-tagged peptide (*n* = 3 mice per treatment group). Error bars represent mean ± SEM. Student’s *t*-test was performed for all relevant figure panels. Significance is indicated with *p < 0.05, **p < 0.01 and ***p < 0.001

We injected 5-week-old mice with either DB or recombinant irisin protein. Quantification of circulating irisin revealed that there was a significant increase in irisin levels in mice injected with irisin (~ 320 ng/ml) when compared to DB-injected mice (~ 213 ng/ml) (Fig. 4a). As expected, injection of irisin increased Ucp1 levels by ~ 4-fold in subcutaneous adipose tissue (Fig. 4b(i), (ii)). Irisin injection did not cause significant difference in food consumption. Body weights increased in both DB- and irisin-injected mice across the 4-week regimen (Fig. 4c). However, the percentage change in body weight was noticeably greater and more rapid in irisin-injected mice when compared to DB-injected mice (Fig. 4c). A significant increase in Quad, M. biceps femoris (BF), M. tibialis anterior (TA), and M. extensor digitorum longus (EDL) muscle weights was seen in mice injected with irisin, when compared to control (Fig. 4d, e). Measurement of grip strength further indicated that the increased muscle mass translated into enhanced grip strength in mice injected with irisin (Fig. 4f). Histological analysis clearly revealed noticeable hypertrophy of myofibers upon irisin injection (Fig. 4g). A marked reduction in the number of myofibers with smaller cross-sectional area (CSA; < 2000 µm^2^), concomitant with a distinct increase in the number of myofibers with larger CSA (>2000 µm^2^), was noted in irisin-injected mice (Fig. 4h).

We did not observe any significant change in the percentage change of the body weights of mice injected with either DB (control) or His-tag peptide (Fig. 4i). Furthermore, there was no significant difference in the hindlimb muscle weights between DB- and His-tag peptide-injected mice (Fig. 4j, k). The histological analysis further confirmed that there was no significant difference in the CSA of muscle fibers between TA muscles extracted from DB- or His-tag peptide-injected mice (Fig. 4l, m).

**Fig. 5:**
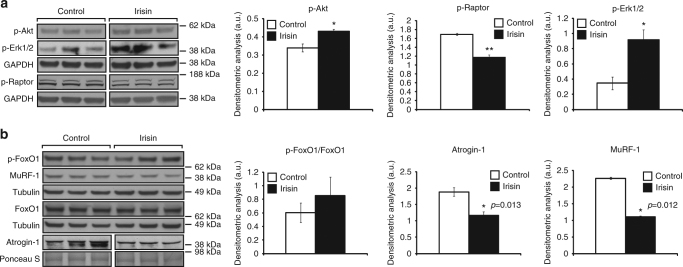
Signalling behind irisin treatment. **a** IB analysis of p-Akt, p-Erk1/2, and p-Raptor protein levels. The levels of GAPDH were assessed as a loading control. Graphs show densitometric analysis of p-Akt (left), p-Raptor (middle), and p-Erk1/2 (right) levels in arbitrary units (a.u.), normalized to GAPDH (*n* = 3 biological replicates). Since p-Akt and p-Erk1/2 IB analysis was performed on the same membrane, the same GAPDH was used to normalize the results. **b** IB analysis of p-FoxO1, MuRF-1, FoxO1, and Atrogin-1 protein levels. The levels of tubulin or Ponceau S were assessed as loading controls. Graphs show densitometric analysis of pFoxO1/FoxO1 (left), Atrogin-1 (middle), and MuRF-1 (right) levels in a.u., normalized to tubulin or Ponceau S (*n* = 3 biological replicates). Since pFoxO1 and MuRF-1 IB analysis was performed on the same membrane, the same tubulin was used to normalize the results. All IBs were performed on proteins collected from 72 h-differentiated myotubes treated with either DB (control) or irisin (1000 ng/ml) for a further 48 h. Where necessary, intervening irrelevant bands were removed from the IBs, which is denoted by a gap between boxes in the figures. Error bars represent mean ± SEM. Student’s *t*-test was performed for all relevant figure panels. Significance is indicated with *p < 0.05 and **p < 0.01

We next analyzed the levels of components of three main pathways that activate protein synthesis; Akt, mTOR and Erk1/2, since the activation of these pathways has been shown to promote skeletal muscle hypertrophy^4^. Treatment of C2C12 myotubes with recombinant irisin protein resulted in a significant increase in the levels of p-Akt (Ser473) (Fig. 5a). As p-Akt in turn activates mTOR, we next analyzed the activity of mTOR by studying the phosphorylation of Raptor. We noted a significant reduction in the phosphorylation of Raptor (Ser792) upon treatment with irisin (Fig. 5a), which is indicative of mTOR activation. Irisin treatment also resulted in a significant increase in the levels of active p-Erk1/2 (Fig. 5a). Taken together these data suggest that irisin promotes skeletal muscle hypertrophy through activating pathways that promote protein synthesis.

As the Akt pathway is closely linked to protein degradation^23^ through ubiquitination, we next analyzed the levels of p-FoxO1, Atrogin-1, and MuRF-1 in irisin-treated myotubes. Subsequent analysis revealed increased levels of inactive p-FoxO1 following treatment with recombinant irisin protein (Fig. 5b). Although the increased p-FoxO1 expression was not statistically significant, we observed a significant reduction in the levels of Atrogin-1 (Fig. 5b) and MuRF-1 (Fig. 5b) following irisin treatment. These data reveal that irisin not only increases anabolism but may also reduce catabolic pathways in skeletal muscle.

### Irisin signals via the IL6 pathway during myogenesis

**Fig. 6:**
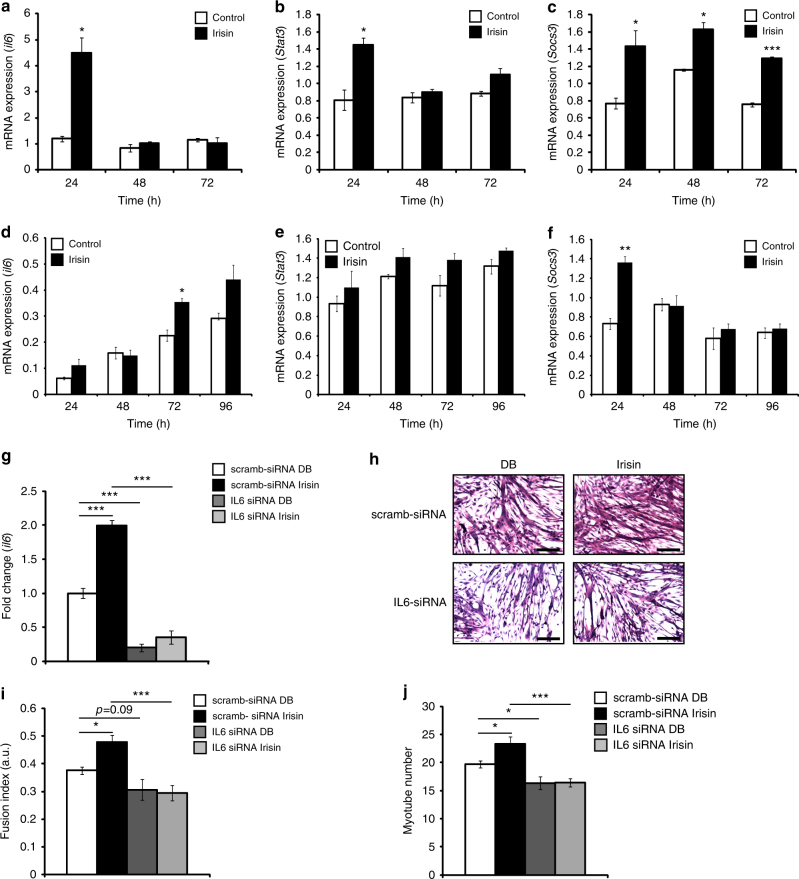
Irisin treatment activates the IL6 pathway. Graphs displaying qPCR analysis of *IL6* (**a**), *Stat3* (**b**), and *Socs3* (**c**) in proliferating myoblasts at 24, 48, and 72 h treated with DB (control) or irisin (1000 ng/ml). Graphs displaying qPCR analysis of *IL6* (**d**), *Stat3* (**e**), and *Socs3* (**f**) in differentiating myoblasts at 24, 48, 72, and 96 h treated with DB (control) or irisin (1000 ng/ml). Graphs displaying qPCR analysis of *IL6* (**g**) at 48 h treated with DB (control) or irisin (1000 ng/ml) in myoblasts transfected with IL6-specific siRNA (IL6 siRNA) or scrambled siRNA-transfected myoblasts (scramb-siRNA). All qPCR graphs show gene expression normalized to *gapdh*. **h** Representative images of H&E-stained IL6 knockdown (IL6 siRNA) and IL6-positive (scramb-siRNA) myoblasts differentiated for 72 h in the presence of DB (control) or irisin (1000 ng/ml). **i** Graph showing quantification of fusion index of IL6-knockdown (IL6 siRNA) and IL6-positive (scramb-siRNA) myoblasts differentiated for 72 h in the presence of DB (control) or irisin (1000 ng/ml). **j** Graph showing quantification of myotube number of IL6-knockdown (IL6 siRNA) and IL6-positive (scramb-siRNA) myoblasts differentiated for 72 h in the presence of DB (control) or irisin (1000 ng/ml) (*n* = 3 biological replicates for all experiments in this figure). Error bars represent mean ± SEM. Student’s *t*-test was performed for all figure panels between **a** and **f**, and two-way ANOVA was performed for all relevant figure panels between **g** and **j**. Significance is indicated with *p < 0.05, **p < 0.01 and ***p < 0.001

Gene expression analysis on RNA from irisin-treated myoblasts revealed that irisin treatment resulted in a significant increase in *IL6*, after 24 h of treatment (Fig. 6a). Consistent with this result, we also observed a significant increase in the expression of *Stat3*, a downstream target of *IL6*, after 24 h of irisin treatment (Fig. 6b). *Socs3*, a negative regulator of the IL6 pathway, is known to be upregulated after IL6 activation^24^. Consistent with this, we observed an upregulation of *Socs3* expression at 24, 48, and 72 h of proliferation with irisin treatment (Fig. 6c). Similar to myoblasts, irisin treatment increased IL6 expression over the time course of differentiation in myotubes (Fig. 6d). Unlike IL6 expression, irisin treatment did not significantly increase Stat3 expression in myotubes. However, an increasing trend in Stat3 expression in response to irisin was observed (Fig. 6e). An increase in Socs3 expression at 24 h of irisin treatment was seen and not thereafter (Fig. 6f).

To further probe the involvement of IL6 in irisin signaling, we knocked down IL6 in differentiating myoblasts and queried if irisin is effective in enhancing myogenesis in the absence of IL6. Treatment with IL6-specific siRNA (IL6 siRNA), but not with control scrambled siRNA (scramb-siRNA), was very effective in the knockdown (~80% downregulation) of IL6 mRNA levels at the 48 h time point (Fig. 6g). While irisin treatment induced significant levels of IL6 in scramb-siRNA-transfected myotubes at 48 h time point, there was a significant inhibition in irisin-mediated induction of IL6 in the presence of IL6 siRNA (Fig. 6g).

To further validate the role of IL6 in the irisin signaling pathway, we performed a differentiation assay on myotubes transfected with IL6 siRNA or control scramb-siRNA and treated with either DB (control) or irisin for 72 h (Fig. 6h). Both fusion index and myotube number showed a significant increase in the scramb-siRNA-transfected + irisin-treated myotubes as compared to the scramb-siRNA-transfected + DB-treated myotubes (Fig. 6i, j), which further validates the results obtained in the differentiation assay (Fig. 2c, d). Knockdown of IL6 in myotubes resulted in impaired differentiation and hence, fusion index (Fig. 6i) and myotube number (Fig. 6j) was reduced in IL6 siRNA-transfected myotubes. It is noteworthy that irisin-treated IL6 siRNA-transfected myotubes also showed a reduction in fusion index (Fig. 6i) and myotube number (Fig. 6j), and a rescue in the phenotype by irisin was not observed. Our results therefore suggest that irisin may partly signal through IL6 to enhance proliferation and differentiation and hence, skeletal muscle hypertrophy.

### Irisin promotes satellite cell activation and expansion

**Fig. 7:**
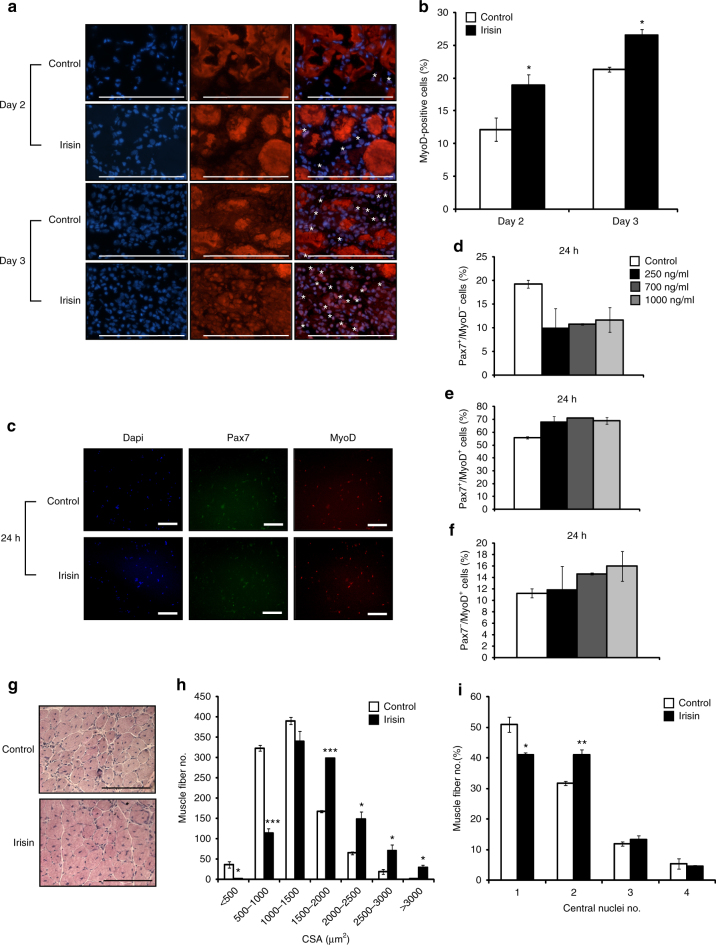
Irisin improves regeneration of skeletal muscle. **a** Representative images of MyoD immunostaining of TA muscle sections from DB (control)- or irisin-injected mice at day 2 and day 3 post notexin injury. Images were captured using a ×40 objective. Scale bar represents 100 μm. Nuclei were counterstained with DAPI. White asterisks denote MyoD-positive cells. Linear brightness and contrast of ‘Merge’ panel was adjusted to visualize MyoD^+^ cells without altering the interpretation of results in any way. **b** Graph displaying the percentage of MyoD-positive cells in TA muscle sections from DB (control)- or irisin-injected mice at day 2 (*n* = 3 mice for both groups) and day 3 (*n* = 2 mice for control group and *n* = 3 mice for irisin-injected group) post notexin injury. **c** Representative images of MyoD and Pax7 immunostaining of primary satellite cell cultures treated with either DB (Control) or irisin (1000 ng/ml) for 24 h. Linear brightness and contrast of “Dapi”, “Pax7”, and “MyoD” panels was adjusted to visualize the positively stained nuclei without altering the interpretation of results in any way. Images were captured using a ×10 objective. Scale bar represents 100 μm. Graphs displaying the percentage of **d** quiescent (Pax7^+^/MyoD^−^), **e** proliferating (Pax7^+^/MyoD^+^), and **f** committed (Pax7^−^/MyoD^+^) satellite cells present following treatment with either DB (control) or increasing concentrations of irisin (250, 700, and 1000 ng/ml) for 24 h (*n* = 2 biological replicates). **g** Representative images of H&E-stained TA muscle at day 10 post notexin injury from mice injected with either DB (control) or irisin. Images were captured using a ×20 objective. Scale bar represents 100 μm. **h** Graphs showing the distribution of TA myofiber cross-sectional area (CSA) and the **i** percentage of myofibers with 1, 2, 3, or 4 centrally placed nuclei at day 10 post notexin injury from mice injected with either DB (control) or irisin (2.5 μg/g of body weight) (*n* = 3 mice for both groups). Error bars represent mean ± SEM. Student’s *t*-test was performed for **b**, **h**, **i**, and one-way ANOVA was performed for **d**–**f**. Significance is indicated with *p < 0.05, **p < 0.01 and ***p < 0.001

We next tested the efficacy of irisin in improving skeletal muscle regeneration. Immunohistochemical analysis on muscle sections with MyoD antibodies (a marker of activated satellite cells) revealed increased percentage of MyoD-positive cells on day 2 and day 3 post-notexin-induced injury in mice injected with irisin, when compared to DB-injected controls (Fig. 7a, b).

To assess if irisin directly activates satellite cells, primary murine satellite cells were cultured, treated with recombinant irisin protein, and subjected to immunocytochemistry to assess the populations of cells positive for Pax7 and MyoD. The percentages of Pax7^+^/MyoD^−^ (quiescent satellite cells), Pax7^+^/MyoD^+^ (proliferating myoblasts), and Pax7^−^/MyoD^+^ (committed myoblasts) cells were assessed in cultures treated with either DB or recombinant irisin protein (Fig. 7c). Treatment with irisin resulted in a marked reduction in the percentage of quiescent satellite cells (Pax7^+^/MyoD^−^) (Fig. 7d), concomitant with an increase in the percentages of proliferating (Pax7^+^/MyoD^+^) (Fig. 7e) and committed myoblasts (Pax7^−^/MyoD^+^) (Fig. 7f). These data suggest that irisin treatment leads to increased satellite cell activation and proliferation.

### Irisin induces hypertrophy during muscle regeneration

Next we assessed the effect of irisin treatment on resulting muscle fiber size and centrally formed nuclei in regenerated skeletal muscle. Subsequent analysis of muscle fiber CSA revealed more myofibers with larger (> 1500 μm^2^) CSA, concomitant with a reduction in myofibers with smaller (< 1500 μm^2^) CSA in regenerated muscle subjected to irisin injection, when compared to DB-injected controls (Fig. 7g, h), thus confirming that irisin injection leads to hypertrophy of skeletal muscle. Consistent with the hypertrophy phenotype seen in the regenerated skeletal muscle, and with the enhanced fusion noted previously (Fig. 2b, d), we observed an increase in the percentage of regenerated myofibers that contained two centrally formed nuclei, with a concomitant decrease in the percentage of myofibers with one centrally formed nuclei, in mice injected with recombinant irisin protein (Fig. 7i).

### Irisin rescues denervation-induced atrophy

**Fig. 8:**
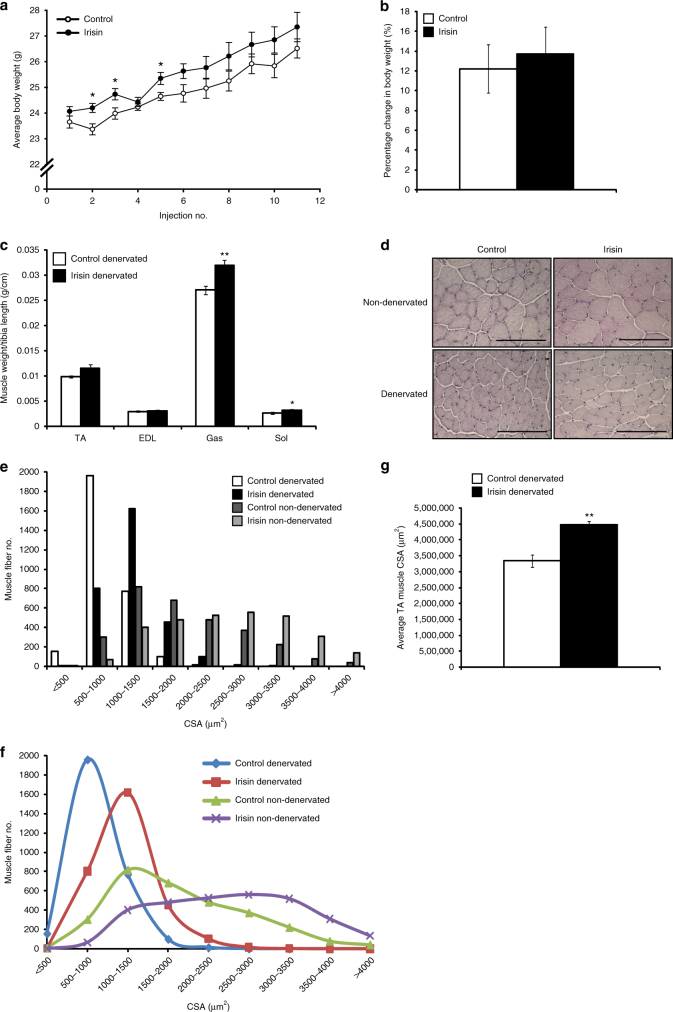
Irisin rescues denervation-induced loss of muscle mass. **a** Graph showing average body weight (g) of mice injected with either DB (control) or irisin (2.5 μg/g body weight) three times a week, 1 week prior to, and 2 weeks post transection of the sciatic nerve. Body weights were measured prior to injection. **b** Graph showing the percentage change in body weight (from initial) in mice injected with either DB (control) or irisin pre and post sciatic nerve transection (*n* = 6 mice for both groups). **c** Graph displaying average TA, EDL, Gas, and Sol muscle weights of denervated mice injected with either DB (control) or irisin pre and post sciatic nerve transection. All hindlimb muscle weights were normalized to tibia length (*n* = 5 mice for DB-injected group and 6 mice for irisin-injected group). **d** Representative images of H&E-stained TA muscle from non-denervated and denervated mice injected with either DB (control) or irisin. **e** Histogram and **f** line graph showing the distribution of TA myofiber CSA in non-denervated and denervated mice injected with either DB (control) or irisin. **g** Graph displaying the average total cross-sectional area of TA muscles from denervated mice injected with either DB (control) or irisin (*n* = 3 mice for both groups). Error bars represent mean ± SEM. Student’s *t*-test was performed for all relevant figure panels. Significance is indicated with *p < 0.05 and **p < 0.01

To investigate if irisin can alleviate muscle atrophy, we utilized a model of denervation-induced atrophy. We observed a significant increase in the average body weight of irisin-injected mice at the initial stage (Fig. 8a). Overall, mice injected with irisin showed a greater percentage increase in body weight (~ 13.7%), when compared to DB-injected mice (~ 12.2%) during the trial (Fig. 8b). However, this difference was not statistically significant. Comparison of muscle weights revealed a significant increase in denervated M. gastrocnemius (Gas) (*p* < 0.01) and soleus (Sol) (*p* < 0.05) muscle weights (Fig. 8c) upon irisin injection. We also noted an increase in the denervated TA muscle after irisin injection, although statistically insignificant. A significant increase in the denervated muscle mass after irisin injection suggested that irisin may have an important role in rescuing skeletal muscle atrophy.

We next performed histological analysis to quantify myofiber CSA in both non-denervated and denervated irisin- and DB-injected mice (Fig. 8d). Subsequent analysis of myofiber CSA confirmed that irisin injection leads to hypertrophy of skeletal muscle fibers (Fig. 8e, f). Consistent with the atrophy model, denervation leads to reduced myofiber CSA (Fig. 8e, f). Due to sciatic nerve injury a considerable increase in the number of smaller muscle fibers (< 1000 µm^2^), concomitant with a decrease in larger muscle fibers (> 1500 µm^2^) was observed in TA muscles of DB-injected mice (Fig. 8e, f). Consistent with the increase in muscle weights observed in irisin-injected mice (Fig. 8c), we noted a significant increase in the numbers of larger muscle fibers, with a concomitant decrease in smaller fibers, in irisin-treated denervated mice, when compared to denervated DB-treated controls (Fig. 8e, f). In agreement with this, the average TA muscle area of irisin-injected denervated mice was significantly (*p* < 0.01) increased, when compared to DB-injected denervated mice (Fig. 8g).

**Fig. 9:**
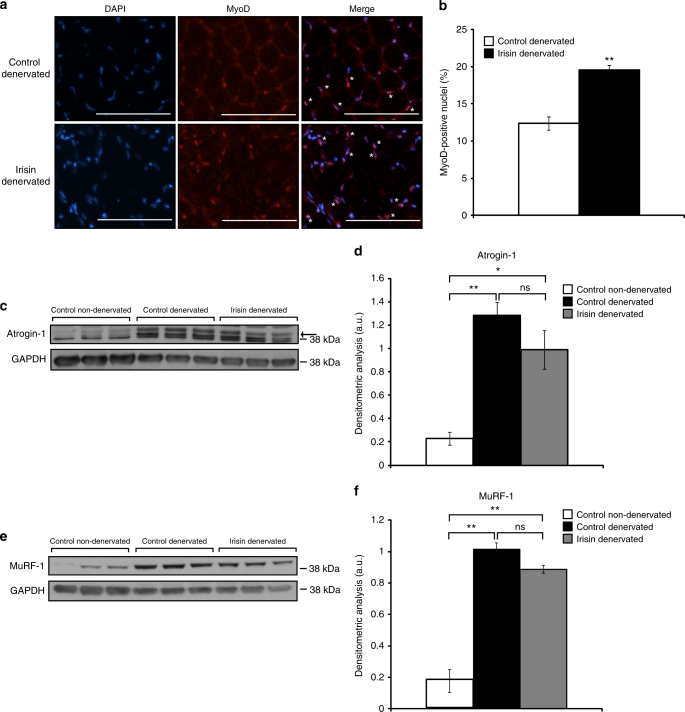
Irisin treatment rescues denervation-induced muscle atrophy. **a** Representative images of MyoD immunostaining of TA muscle sections from denervated mice injected with either DB (control) or irisin. Images were captured using a ×20 objective. Scale bar represents 100 μm. Nuclei were counterstained with DAPI. White asterisks denote MyoD-positive cells. Linear brightness and contrast of ‘Merge’ panel was adjusted to visualize MyoD^+^ nuclei without altering the interpretation of results in any way. **b** Graph displaying the percentage of MyoD-positive cells in TA muscle sections from denervated mice injected with either DB (control) or irisin pre and post sciatic nerve transection. **c** IB analysis of Atrogin-1 protein levels in Gas muscle from non-denervated mice injected with DB (control) and denervated mice injected with either DB (control) or irisin. The levels of GAPDH were assessed as a loading control. Arrow indicates Atrogin-1 band. **d** Graph shows densitometric analysis of Atrogin-1 levels in arbitrary units (a.u), normalized to GAPDH. **e** IB analysis of MuRF-1 protein levels in Gas muscle from non-denervated mice injected with DB (control) or denervated mice injected with either DB (control) or irisin. The levels of GAPDH were assessed as a loading control. **f** Graph shows densitometric analysis of MuRF-1 levels in a.u, normalized to GAPDH (*n* = 3 biological replicates). Error bars represent mean ± SEM. Student’s *t*-test was performed for **b** and one-way ANOVA was performed for **d** and **f**. Significance is indicated with *p < 0.05 and **p < 0.01

Skeletal muscle injury leads to activation of satellite cells, which function to repair and regenerate skeletal muscle tissue. As such, we next performed immunocytochemistry on TA muscle sections to determine the numbers of nuclei positive for MyoD (a marker of activated satellite cells) (Fig. 9a). Subsequent quantification revealed a significant increase in the numbers of MyoD-positive nuclei, consistent with increased satellite cell activation, in irisin-injected denervated TA muscle, when compared to respective DB-injected controls (Fig. 9b). These data suggest that irisin treatment results in increased activation of satellite cells during denervation-induced skeletal muscle atrophy.

Atrogin-1 and MuRF-1 are two E3 ubiquitin ligases that are used as reliable markers for protein degradation during atrophic conditions, including denervation^25^. Therefore, we next measured the protein levels of both Atrogin-1 and MuRF-1 in Gas muscle isolated from non-denervated, denervated DB-injected mice, and denervated irisin-injected mice. As expected, we observed a significant increase in Atrogin-1 (Fig. 9c, d) and MuRF-1 (Fig. 9e, f) protein levels in response to denervation-induced skeletal muscle atrophy. However, injection of irisin resulted in a notable reduction in Atrogin-1 (Fig. 9c, d) and MuRF-1 (Fig. 9e, f) protein levels in denervated muscle, when compared to muscle isolated from denervated, DB-injected mice. These data suggest that irisin treatment leads to reduced expression of key markers of skeletal muscle wasting during denervation-induced muscle atrophy.

## Discussion

Irisin is a novel myokine^10^ that has been shown to induce browning of white adipocytes. However, the endocrine/paracrine function of this myokine on skeletal muscle during postnatal myogenesis is still uncharacterized. We reasoned that irisin could improve myogenesis and/or induce hypertrophy of skeletal muscle since irisin is an exercise-induced factor. Our findings are consistent with this hypothesis and the extended results here provide an exemplification of the therapeutic benefits of irisin in improving muscle healing after injury and alleviating atrophy of skeletal muscle in mouse models.

For this study we have used *Escherichia coli*-purified recombinant irisin protein with low levels of endotoxin. It is also important to note that after irisin injection, mice did not show any adverse reaction or notable change in movement, food, or water intake. Furthermore, no significant pathology or toxicity was observed in any major organ system. No signs of inflammation or fluid retention in any part of the body was seen. Hence, we believe the recombinant protein was non-toxic.

As expected, the recombinant irisin protein used in this study was able to increase the expression of *Ucp1* in white adipocyte cultures (Fig. 1b, c), as well as increased Ucp1 protein expression levels in subcatenous adipose tissue (Fig. 4b) confirming that it is biologically active. Recently, it has been reported that human irisin is glycosylated^26^. Since *E. coli*-expressed proteins are not glycosylated, it is possible that the recombinant irisin protein that we have used may not be fully active^26^.

We used this validated protein to monitor the effect of irisin on skeletal muscle. Since irisin is a secreted myokine, which is shown to be a systemic factor^27^, we either injected mice with recombinant irisin protein or treated myogenic cultures with recombinant irisin protein, to monitor the biological effect of irisin on skeletal muscle both in vitro and in vivo. Several lines of evidence suggest that irisin is a pro-myogenic factor. Microarray analysis and subsequent qPCR validation revealed that several negative regulators of myogenesis, such as *sox8*, *heyL*, and *ogn* were downregulated, while pro-myogenic factors, such as *sema3f*, *il6*, *shq1*, *il7*, *erpina3g*, *bmp4*, *tnfrsf11b*, and *agxt2*, were upregulated in response to irisin treatment (Fig. 1 and Table 1). It is also interesting to note that irisin treatment led to a significant increase in a number of genes associated with exercise, which is consistent with the hypothesis that irisin is an exercise-induced myokine that may impart benefits of exercise on skeletal muscle. Furthermore, exogenous addition of irisin improved myogenic differentiation in myoblasts, as quantitative analysis revealed increased myotube number and myotube fusion index in response to irisin treatment. It is noteworthy that murine irisin was also able to induce myogenesis in human primary myoblast cultures. Although we have not directly addressed the issue whether human irisin can induce myogenesis in human myoblasts, the preliminary results presented here allude to the existence of a receptor that can bind to irisin-like molecule on human myoblasts. Further work is required to purify recombinant human irisin and fully characterize its bioactivity on human myoblasts.

The enhanced myogenesis in C2C12 cultures due to irisin treatment appears to be due to increased fusion of myoblasts, as irisin upregulated the expression of *myomaker* ^28^ and *caveolin-3* ^29^, two genes previously shown to be required for myoblast fusion. Consistent with what we observed in vitro, when irisin protein was injected during muscle regeneration, we observed an increase in MyoD-positive proliferating myoblasts at day 2 and day 3 post injury. Our analysis further indicated that irisin treatment enhanced the pool of proliferating (Pax7^+^/MyoD^+^) and fusion competent (Pax7^−^/MyoD^+^) myoblasts in primary myoblast cultures. Following regeneration we also observed an increase in myofiber CSA along with an increase in the numbers of myofibers that contain two centrally formed nuclei. These data support that irisin promotes improved fusion of satellite cell-derived myoblasts, leading to hypertrophy of skeletal muscle. Therefore, taken together, in vitro and in vivo results reveal that irisin could potentially improve myogenesis through not only increasing the fusion competent myoblast pool but also through enhancing myoblast fusion capacity.

Both microarray analysis and subsequent real-time PCR analysis confirmed that irisin is a potent inducer of *IL6*. It has been previously shown that IL6 levels are induced during myogenic differentiation^30^, and that absence of *IL6* mRNA reduced myogenic differentiation while overexpression of *IL6* mRNA enhanced myogenic differentiation^30^. Much like irisin, IL6 is secreted by skeletal muscle during exercise^31^ and IL6 has also been identified as an essential regulator of skeletal muscle hypertrophy via satellite cell activation^32^. Furthermore, absence of *IL6* reduced muscle hypertrophy and satellite cell proliferation in a Stat3-dependent manner^32^. All these circumstantial evidence made IL6 a very good downstream target of irisin. Consistent with this theory, we observed that irisin is a potent inducer of *IL6* in both myoblasts and myotubes, and that knocking down of *IL6* resulted in impaired ability of irisin to induce myogenesis. Therefore, we propose that at least in part, irisin signals via IL6 to promote myogenesis.

Since irisin is an exercise mimetic, we investigated if irisin can induce an exercise phenotype in mouse muscles. Wild-type mice injected with irisin showed increased body weights, which could be attributed to increased skeletal muscle mass. Irisin injection further resulted in improved muscle strength, which was reflected by an increase in grip strength. Subsequent CSA analysis revealed a significant hypertrophy phenotype in muscle of mice injected with irisin. Taken together, these observations are consistent with typical changes noted in response to resistance exercise. However, none of the effects of irisin on skeletal muscle in vivo could be recapitulated with a control peptide or vehicle control (DB). Hypertrophy of skeletal muscle could occur via three mechanisms: increased protein synthesis^4, 5^; reduced protein degradation^25, 33^; and increased satellite cell activation^34^. Irisin injection resulted in a significant increase in the numbers of activated myoblasts and further enhanced myoblast fusion during regeneration. Furthermore, we observed increased IL6, p-Akt, and p-Erk1/2 levels, concomitant with reduced p-Raptor levels upon treatment with irisin, which are indicative of increased protein synthesis. In addition to increased protein synthesis we have further noted reduced protein degradation upon irisin treatment. Specifically, we noted increased expression of haptoglobin upon irisin treatment. Interestingly, previous work has revealed that absence of haptoglobin resulted in skeletal muscle atrophy, both through upregulating atrophy markers, such as the E3 ligases Atrogin-1, and MuRF-1, and via downregulating protein synthesis^35^. Thus, it is interesting to surmise that in addition to promoting protein synthesis, irisin may also prevent excess skeletal muscle protein turnover through enhancing haptoglobin levels. In agreement with reduced protein turnover, we observed reduced Atrogin-1 and MuRF-1 levels upon irisin treatment. The levels of Atrogin-1 and MuRF-1 are regulated by FoxO transcription factors and hence we expected to observe a significant increase in the phosphorylation status of FoxO1, which equates to inactivation of FoxO1, upon irisin treatment. Although we see a trend of increased levels of p-FoxO1, the increase was not statistically significant. This suggests that irisin may decrease the levels of Atrogin-1 and MuRF-1 through additional mechanisms. It is quite possible that regulation of additional upstream mediators of Atrogin-1 and MuRF-1 expression, such as nuclear factor-κB^36^, myostatin/transforming growth factor-β signaling^33^, and p38 MAPK^37^ may play a role in irisin regulation of protein degradation. Taken together these data suggest that irisin promotes skeletal muscle hypertrophy by shifting the balance toward increased protein synthesis and reduced protein breakdown.

To further prove the pro-myogenic function of irisin we next assessed the utility of irisin injection in overcoming the severe atrophy associated with denervation. Sciatic nerve transection leads to denervation of skeletal muscle and severe skeletal muscle atrophy through activation of components of the ubiquitin proteosome pathway, including Atrogin-1 and MuRF-1. Previous studies have shown that exercise is able to rescue skeletal muscle atrophy through promoting an increase in lean muscle mass^38^. Here we show that injection of irisin was able to partially rescue the atrophic phenotype observed in a mouse model of denervation-induced atrophy. Mechanistically, we propose that irisin injection leads to increased activation of satellite cells (Fig. 7a–c, e, f) and reduces protein degradation by reducing Atrogin-1 and MuRF-1 (Fig. 9c–f) resulting in a partial rescue of atrophy.

In the present study we have assessed the function of irisin in regulating muscle growth in mice. For this purpose we have used murine irisin to exemplify that irisin induces hypertrophy and also promotes myogenesis. Although we were able to partially recapitulate the effects of resistance exercise such as hypertrophy on skeletal muscle through irisin treatment, it is important to highlight that we did not compare the extent of hypertrophy between resistance exercise and irisin-induced hypertrophy. Furthermore, in the light of recent confirmation of the presence of human irisin protein in circulation, and the fact that irisin levels are increased in humans in response to exercise^18^, further work needs to be undertaken to confirm the biological effects of human irisin in human skeletal muscle.

## Methods

### Expression and purification of recombinant irisin protein

Recombinant M-irisin was expressed and purified using the pET expression system (Novagen). M-irisin cDNA (encoding aa 29–140 of FNDC5) was PCR-amplified and cloned into pET-16b expression vector in frame with 10 histidine residues using standard molecular biology techniques. The resulting construct was transformed into *E. coli* strain BL21 (Agilent Technologies, USA) and was cultured in Lennox broth (LB) containing ampicillin (100 mg/l) overnight as starter culture. The starter culture was diluted into 4 l of LB medium plus ampicillin and was further grown to an OD of 0.8 at 595 nm. Bacteria were then induced to produce histidine-tagged irisin through addition of 1 mM isopropyl-1-thio-β-d-glalactopyranoside for 2.5 h. Bacteria were harvested by centrifugation, resuspended in 100 ml of binding buffer (50 mM Tris-HCl (pH 8.0), 200 mM NaCl, 10% glycerol, and 20 mM imidazole) and sonicated. The lysate was centrifuged for 30 min at 10,000×*g* and the supernatant was collected and run through a Ni-NTA agarose affinity column (Qiagen) for recombinant irisin purification, according to the manufacturer’s protocol. Fractions containing recombinant irisin protein eluted from the Ni-NTA agarose column were pooled and dialyzed against three changes of DB (50 mM Tris-HCl (pH 8.0), 500 mM NaCl, and 10% glycerol) for a period of 3 h. Endotoxin level in recombinant irisin protein was estimated by ToxinSensor Chromogenic LAL kit (GenScript, USA) according to the manufacturer’s protocol.

### Animals

Wild-type C57BL/6J male mice used in the study were obtained from either Nanyang Technological University (NTU) Animal house, A*STAR Biological Resource Centre, Singapore or InVivos, Singapore. All experiments were performed on 5- to 8-week-old mice according to the Institutional Animal Care & Use Committee (IACUC)-approved protocols. IACUC granted permission to perform all animal experiments in this manuscript. All mice were maintained on standard chow diet at a constant temperature of 20 °C under an artificial 12 h light and 12 h dark cycle with ad libitum access to water at the NTU animal house. To assess pro-myogenic function of irisin, 5-week-old C57BL/6J mice were injected with 2.5 μg/g body weight of irisin twice a week intraperitoneally (IP) for 4 weeks and control animals were injected with DB, vehicle control. For control peptide animal trial, we used N-terminal His-tag of recombinant irisin fusion protein coded by the pET-16b vector. The 20-aa-long peptide (MGHHHHHHHHHHSSGHIEGR) was synthesized by Merck, Sigma-Aldrich. Similar to the irisin previous animal trial, 5-week-old C57BL/6J mice were injected with 2.5 μg/g body weight of His-tag peptide thrice a week IP for 4 weeks and control animals were injected with DB as vehicle control. Body weight and food consumption were measured during animal trial. Following the trial, mice were sacrificed by CO_2_ asphyxiation and various skeletal muscle tissues were collected for further experiments. TA muscle tissue was embedded in optimal cutting temperature compound (OCT) (Sakura Finetek, USA), frozen in liquid nitrogen-cooled isopentane, and stored at −80 °C for subsequent sectioning and staining.

Injury of resting muscle was performed through injection of notexin. A unit of 100 µg of notexin powder (Latoxan, L8104) was dissolved in 1 ml of autoclaved saline and diluted to 10 µg/ml before injection. Mice were anesthetized through IP injection of a mixture of ketamine and xylazine at 0.1 ml/10 g of body weight. A volume of 15 µl of 10 µg/ml notexin was injected along the longitudinal axis of left TA muscles of 6-week-old C57BL/6J wild-type mice^39^. The right TA muscle was used as an uninjured control. Mice were injected IP with DB or recombinant irisin protein (2.5 µg/g body weight) three times a week, 1 week prior to the injury, and also following notexin injury for the duration of the trial. TA muscles were collected and OCT-embedded as mentioned above from mice at days 1, 2, 3, and 10 post-notexin-induced injury.

In order to investigate if irisin reduces the atrophy of skeletal muscle, 8-week-old C57BL/6J male mice were anesthetized through IP injection of a mixture of ketamine and xylazine at 0.1 ml/10 g of body weight. Hair on the hindlimb was shaved using an electric shaver. A small incision was made and the sciatic nerve of the left leg was isolated and cut using surgical scissors. The skin was glued to close the wound. The contralateral (right) leg was used as the uninjured control. Mice were IP-injected with either DB or irisin (2.5 µg/g body weight) for 1 week prior to injury and 2 weeks post injury, at a frequency of three times a week. Following conclusion of the trial, mice were sacrificed by CO_2_ asphyxiation and various skeletal muscle tissues were collected for further experiments. TA muscle tissue was embedded in OCT (Sakura Finetek), as mentioned above for subsequent sectioning and staining.

### Detection of irisin in serum

Blood collected from mice injected with DB or irisin twice a week for 4 weeks were incubated at room temperature (RT) for 30 min. Blood samples were then centrifuged at 1500×*g* for 15 min at 4 °C and serum was isolated. Irisin levels in the serum were quantified using a commercial ELISA kit following the manufacturer’s protocol (Irisin ELISA kit EK-067−52, Phoenix Pharmaceuticals, Inc., USA) using spectrophotometry at a wavelength of 450 nm. The detection range of the kit is 0.1–1000ng/ml.

### Muscle fiber CSA measurement

Transverse sections (10 µm) were cut from the mid-belly region of OCT-embedded TA muscle using a cryostat (Leica CM 1950, Germany) and sections were stained with Gill’s hematoxylin followed by 1% eosin (Merck; H&E). TA muscle sections were photographed and tiled using the Leica CTR 6500 microscope equipped with the Leica DFC 420 camera and Image Pro Plus software (Media Cybernetics, Bethesda, MD, USA). The CSA of 200 muscle fibers from 5 random fields (1000 fibers per section per mouse) were analyzed (*n* = 3 mice per treatment group).

### Immunohistochemical staining of muscle sections

Muscle sections were fixed with 4% paraformaldehyde in phosphate-buffered saline (PBS) (pH 7.4) for 10 min and then permeabilized with 0.1% Triton X-100 for 10 min. Sections were blocked with 5% normal sheep serum (NSS), 5% normal goat serum (NGS), and 0.35% Carrageenan lambda (Cλ) in PBS for 1 h at RT and incubated with anti-MyoD (SC-304, 1:50) primary antibody overnight at 4 °C. Sections were then incubated with secondary antibody (Alexa Fluor 568 goat anti-rabbit 1:300) for 1 h at RT. Sections were counterstained with 4,6-diamidino-2-phenylindole (DAPI) (1:1000) for 5 min in PBS and mounted using Prolong Gold Anti-Fade (Thermo Fisher Scientific, USA). Stained samples were analyzed and MyoD-positive nuclei were counted, with the number of MyoD-positive nuclei expressed as a percentage of total DAPI-positive nuclei. Images from 20 random microscopic fields for each treatment were analyzed per mouse using the Leica CTR 6500 microscope equipped with the Leica DFC 420 camera and Image Pro Plus software (Media Cybernetics).

### Cell culture

American Type Culture Collection (ATCC) murine C2C12 myoblasts (Yaffe and Saxel, 1977)^40^ were maintained in proliferation medium, consisting of Dulbecco’s modified Eagle’s medium (DMEM) (Invitrogen, Carlsbad, CA, USA), 10% fetal bovine serum (HyClone, Thermo Scientific, USA), and 1% penicillin/streptomycin (P/S) (Gibco, Invitrogen). 36C15Q primary human myoblasts were a gift from Drs. Vincent Mouly and Gillian Butler-Browne from the Institut de Myologie and were maintained in proliferation medium, consisting of DMEM, 20% fetal bovine serum, 10% horse serum (HS), 1% chick embryo extract (CEE), and 1% P/S.

C2C12 myoblasts were seeded at a density of 1000 cells/well in 96-well plates in proliferation media and incubated at 37 °C in 5% CO_2_ incubator. Proliferating myoblasts were treated with DB or recombinant irisin protein (250, 1000, and 2000 ng/ml) and fixed at 0, 24, 48, and 72 h. The time when myoblasts were attached was taken as 0 h. Myoblasts were fixed with 200 µl of fixative solution per well (10% formaldehyde (Sigma-Aldrich, USA) and 0.9% NaCl). Plates were wrapped in aluminum foil and stored at RT till all plates were fixed. Proliferation was assessed using the methylene blue photometric end point assay^41^ where absorbance at 655 nm is directly proportional to the total cell number. Each treatment for each time point was conducted in 16 wells.

C2C12 myoblasts were seeded on plastic thermanox cover slips (Nalge Nunc International, USA) in 24-well-plates at a density of 15,000 cells/cm^2^ while 36C15Q primary human myoblasts were seeded at a density of 25,000 cells/cm^2^. After overnight cell attachment myoblasts were differentiated with low-serum differentiation medium (DMEM with 2% HS (Hyclone, Thermo Scientific, USA) and 1% P/S) at 37 °C, 5% CO_2_ and were treated with either DB or recombinant irisin protein (250 or 1000 ng/ml) during differentiation. After 0, 24, 48, 72, and 96 h of differentiation, myotubes were fixed with 70% ethanol, formalin, and acetic acid in a 20:2:1 ratio and washed with 1 ml of PBS three times before H&E staining. Images of non-overlapping areas from three coverslips were taken in a bright-field microscope per time point (Leica CTR 6500 microscope equipped with the Leica DFC 420 camera). To analyze myotube number 10 random images were obtained per coverslip at a ×10 magnification and the total number of myotubes was counted in each image. Fusion index was measured by dividing the number of nuclei found within the myotubes by the total number of nuclei in each image.

Primary cultures were isolated from mouse hindlimb muscle tissue. Muscle tissues collected from wild-type mice were minced well in PBS and centrifuged for 10 min at 3000 r.p.m. to remove the PBS. Minced muscles were resuspended in filter sterilized 0.2% Collagenase Type IA (Sigma-Aldrich, USA) in DMEM and incubated at 37 °C for 90 min on a shaker at 70 r.p.m. (slow shaking). The suspension was centrifuged at 3000 r.p.m. for 10 min. The pellet was resuspended in 12 ml of PBS and strained with a 100 µm nylon cell strainer (BD Falcon, USA). The filtered suspension was further centrifuged at 3000 r.p.m. for 10 min and the pellet was resuspended in warm satellite cell proliferation media (DMEM, 20% FBS, 10% HS, 1% CEE (Biomed Diagnostics, Singapore), and 1% P/S). The suspension was pre-plated on a non-coated 10 cm dish and left at 37 °C and 5% CO_2_ for 3 h for removal of fibroblasts from the culture. Subsequently, the “fibroblast-free” cell suspension was plated onto a 10 cm cell culture dish coated with 10% Matrigel (Corning, USA) and incubated overnight at 37 °C, 5% CO_2_. After overnight attachment, primary myoblasts were trypsinized and seeded at a density of 10,000 cells/well in 8-well chamber slides (Thermo Fisher Scientific)^39^. Primary myoblasts cultures were treated with recombinant irisin protein (250, 700, or 1000 ng/ml) and were fixed at 24 h post irisin treatment with 4% paraformaldehyde in PBS for 15 min. Fixed primary myoblasts were then treated with 0.1% Triton X-100 for 10 min to permeabilize the cells. Cells were blocked with 5% NSS, 5% NGS, and 0.35% Cλ in PBS for 1 h at RT and incubated with primary antibodies, anti-Pax7 (DSHB AB-528428) (1:100), and anti-MyoD (Santa Cruz, SC-304) (1:100) overnight at 4 °C. After washing, myoblasts were incubated with secondary antibody (sheep anti-mouse IgG biotinylated (1:300)) for 1 h at RT. Finally, cells were treated with tertiary antibody (streptavidin-conjugated Alexa Fluor 488 (1:400) and goat anti-rabbit Alexa Fluor 594 (1:300)) for 1 h at RT in the dark. DAPI staining was performed for 5 min (1:1000 dilution in PBS) and slides were mounted using Prolong Gold Anti-Fade. Stained samples were analyzed for the numbers of Pax7^+^ and MyoD^+^ myoblasts. Images from 20 random microscopic fields per treatment were analyzed using the Leica CTR 6500 microscope (×10 objective) equipped with the Leica DFC 420 camera and Image Pro Plus software (Media Cybernetics).

IL6 targeting siRNA (IL6 siRNA) or control non-targeting siRNA (scramb-siRNA) (Dharmacon, Inc., USA) were transfected into C2C12 myoblasts for 24 h using DharmaFECT 1 transfection reagent according to the manufacturer’s protocol. Following transfection, C2C12 myoblasts were switched to differentiation medium and treated with either DB or irisin for 72 h and stained with H&E for histological analysis. In order to investigate if irisin signals through IL6, IL6 siRNA and control siRNA (IL6 siRNA) were transfected with DharmaFECT 1 transfection reagent for 24 h into myoblasts and differentiated for 48 h in differentiation medium. RNA was extracted from myotubes and real-time PCR was used to measure gene expression changes as mentioned above.

### Human adipose-derived stem cells

Details regarding establishment of the human primary ADSCs have been described previously^42^. The collection of white adipose tissue biopsies and generation of human primary ADSC cultures was approved by the Domain Specific Review Board (#2013/00171) of National University Hospital, Singapore. Approximately 1 g of white adipose tissue was digested with type IA collagenase (1 mg/ml) in bovine serum albumin (20 mg/ml) for 90 min at 37 °C with shaking (120 r.p.m.). The suspension was centrifuged for 10 min at 800×*g* and the cell pellet was suspended in 9 ml of red blood cell lysis buffer and incubated for 10 min. The cells were centrifuged again for 10 min at 800×*g* and resuspended in growth medium, consisting of DMEM/F-12 (Gibco, Thermo Fisher Scientific), supplemented with 20% FBS and 1% P/S, and subsequently filtered through a 100 µm nylon cell strainer. The filtrate was further centrifuged for 10 min at 800×*g* and the pellet was resuspended with hADSC growth medium. To induce adipogenic differentiation, hADSC cells were seeded on 10 cm dishes coated with 0.2% gelatin and grown in DMEM/F12, 10% FBS, and 1% P/S. Cells were grown to confluence and left for 2 days before treatment with induction medium (DMEM/F-12, 10% FBS, 1% P/S, 0.5 mM IBMX, 1 µM dexamethasone, 200µM indomethacin and 58µg/ml insulin) containing DB or irisin (4 μg/ml) for 14 days. The induction medium was then replaced with insulin medium (DMEM/F-12, 10% FBS, 1% P/S, and 0.01 mg/ml insulin) for another 7 days in the presence of either DB or irisin (4 μg/ml)

The murine embryonic fibroblast cell line 3T3L1 (ATCC-CL173), which can be induced to differentiate into adipocytes in culture, was also used in this study. Adipogenic differentiation was performed as per the previously described protocol^43^. 3T3L1 cells were maintained in growth medium consisting of DMEM + GlutaMAX, 10% bovine calf serum (Gibco, Thermo Fisher Scientific), and 1% P/S. To induce adipogenic differentiation, 3T3L1 cells were seeded on 10 cm dishes coated with 0.2% gelatin and grown in DMEM with 2 mM l-glutamine, 10% FBS, and 1% P/S. Cells were grown to confluence for 72 h and kept at that state for another 48 h to arrest cell division. 3T3L1 cells were then treated with induction medium (DMEM + GlutaMAX, 10% FBS, 1% P/S, 0.5 mM IBMX, 1 µM dexamethasone, and 0.01 mg/ml insulin) containing DB or irisin (0.5 μg/ml) for a further 48 h. The induction medium was then replaced with insulin medium (DMEM + GlutaMAX, 10% FBS, 1% P/S, and 0.01 mg/ml insulin) for another 48 h in the presence of either DB or irisin (0.5 μg/ml).

### Western blot analysis

A unit of 50 mg of muscle tissue was homogenized in RIPA buffer (1× PBS, 1% IGEPAL CA-630 (v/v), 0.1% sodium dodecyl sulfate (SDS) (w/v), 0.5% sodium deoxycholate (w/v)), and 50 mM sodium fluoride) using the Tissue Lyser II instrument (Qiagen, USA) at 30 Hz (3 × 1 min). For in vitro experiments, C2C12 cells were re-suspended in protein lysis buffer (1 M Tris-HCl (pH 7.5), 5 M NaCl, 0.5 M EDTA, IGEPAL CA-630, protease inhibitor, and MilliQ water). C2C12 cells in lysate buffer were syringed 20 times with a 1 ml syringe and 26 gauge needle. Subsequently, both muscle homogenates and C2C12 cells were centrifuged at 12,000 r.p.m. for 10 min at 4 °C. Protein estimations were done using Bradford’s assay reagent. For all western blot analyses, 5–20 µg of protein was resolved by SDS-PAGE (NuPage 4–12% gradient Bis-Tris pre-cast polyacrylamide gels, Invitrogen) and transferred to nitrocellulose membrane by electroblotting. The following primary antibodies were used for western blotting: MyoD (1:500, sc-304, Santa Cruz); myogenin (1:400 sc-576, Santa Cruz); p21 (1:200, 556430, BD Pharmingen); MHC (MF-20) (1: 400, DSHB); Akt1/2/3 (1:500, sc-8312, Santa Cruz); p-Akt (S473) (1:500, sc-7985, Santa Cruz); Erk1/2 (1:500, sc-292838, Santa Cruz); p-Erk (1:500, sc-16982, Santa Cruz); Raptor (1:500, 4978, Cell Signalling); p-Raptor (S792) (1:500, 2083, Cell Signalling); p-FoxO1 (1:500, sc-101681, Santa Cruz); FoxO1 (1:500, sc-11350, Santa Cruz); MAFbx/Atrogin-1 (1:500, PAB15627, Abnova); MuRF-1 (1:1000, gift from Regeneron); GAPDH, 1:10,000 dilution of purified mouse monoclonal anti-GAPDH antibody (G9545, Sigma-Aldrich); and α-tubulin, 1:10,000 dilution of purified mouse monoclonal antibody (T5168, Sigma-Aldrich). Uncropped scans of western blots are shown in Supplementary Figs. 1–5.

### Quantitative real-time PCR

Total RNA was isolated from C2C12 myotubes using TRIzol reagent (Invitrogen), according to the manufacturer’s instructions. RNA integrity was monitored by RNA electrophoresis. A unit of 1 μg of total RNA was used to synthesize cDNA according to the iScript cDNA Synthesis kit protocol (Bio-Rad). qPCR reactions were carried out in triplicates using SsoFast EvaGreen Supermix (Bio-Rad) and the CFX96 Real-Time System (Bio-Rad). All reactions were performed using the following thermal cycle conditions: 95 °C for 3 min, followed by 40 cycles of a three-step reaction, denaturation at 95 °C for 10 s, annealing at 60 °C for 10 s, elongation at 72 °C for 20 s, followed by a melting curve from 65 to 95 °C in 10 s increment of 0.5 °C. Gene expression fold change was calculated using the ΔΔC_t_ method, normalized against the expression of *Gapdh*. Primers stocks (100 μM; Sigma-Aldrich, Singapore) were diluted to 2.5 μM prior to qPCR. The sequences of the primers used in this study are provided below: *Caveolin 3-F*: 5ʹ-GA TCT GGA AGC TCG GAT CAT-3ʹ, *Caveolin 3-R*: 5ʹ-TCC GCA ATC ACG TCT TCA AAA T-3ʹ; *Myomaker-F*: 5ʹ-GAC AGT GAG CAT CGC TAC CA-3ʹ, *Myomaker-R*: 5ʹ-GTT CAT CAA AGT CGG CCA GT-3ʹ; *GAPDH-F*: 5ʹ-ACA ACT TTG GCA TTG TGG AA-3ʹ, *GAPDH-R*: 5ʹ-GAT GCA GGG ATG ATG TTC TG-3ʹ; *Sox8-F*: 5ʹ-CTG TGG CGC TTG CTG AGT-3ʹ, *Sox8-R*: 5ʹ-CGG CCA GTC TTC ACA CTC TT-3ʹ; *Heyl-F*: 5ʹ-TGC CTT TGA GAA ACA GGC T-3ʹ, *Heyl-R*: 5ʹ-AGG CAT TCC CGA AAC CCA AT-3ʹ; *Haptoglobin-F*: 5ʹ-TTC TAC AGA CTA CGG GCC GA-3ʹ, *Haptoglobin-R:* 5ʹ-CCC ACA CAC TGC CTC ACA TT-3ʹ; *IL6-F*: 5ʹ-GGG ACT GAT GCT GGT GAC AA-3ʹ, *I* *L6-R*: 5ʹ-TGC CAT TGC ACA ACT CTT TTC T-3ʹ; *CXCL1-F*: 5ʹ-CCG AAG TCA TAG CCA CAC TCA-3ʹ, *CXCL1-R*: 5ʹ-GTG CCA TCA GAG CAG TCT GT-3ʹ; *Ptx3-F*: 5ʹ-CCC GCA GGT TGT GAA ACA G-3ʹ, *Ptx3-R*: 5ʹ-TAG GGG TTC CAC TTT GTG CC-3ʹ; *Stat3-F*: 5ʹ-GGC ACC TTG GAT TGA GAG TC-3ʹ, *Stat-3-R*: 5ʹ-ACT CTT GCA CCA ATC GGC TA-3ʹ; Socs3-F: 5ʹ-AAC CCT CGT CCG AAG TCC C-3ʹ, *Socs3-R:* 5ʹ-TTC CGA CAA AGA TGC TGG AG-3ʹ.

### Microarray analysis

C2C12 myoblasts were seeded at a density of 25,000 cells/cm^2^ in a 10 cm cell culture dish and differentiated with low-serum differentiation medium at 37 °C, 5% CO_2_ for 72 h and further treated with either DB or recombinant irisin protein (1000 ng/ml) for 6, 12, 24, and 48 h. Myotubes were resuspended in 2 ml of TRIzol for each 10 cm dish and RNA isolation was performed according to the manufacturer’s protocol. Purified isolated RNA was then subjected to Illumina bead array sequencing and data analysis, as per in-house protocols (Sciencewerke, Singapore). Only genes that were upregulated or downregulated by ≥1.5-fold were considered significant. For genes that had multiple probes (with different accession number), only one probe with the highest upregulation or downregulation was taken into consideration.

### Statistical analysis

Statistical analysis was performed using two-tailed Student’s *t*-test to compare differences between two groups. F-test was performed prior to Student’s *t*-test to determine equal variance between the two groups. Variance between two groups was considered equal when F-value was smaller than F-critical (type 2 error) and unequal if F-value was larger than F-critical values (type 3 error). One-way analysis of variance (ANOVA) was performed for all experiments that require a comparison between three or more groups within one categorical variable followed by a Tukey’s post hoc test. For experiments requiring the analysis of two categorical variables that could influence the numerical output, two-way ANOVA was performed followed by Scheffe’s post hoc test. For all experiments, results were considered significant at *p* < 0.05 (*), *p* < 0.01 (**) and *p* < 0.001(***). Data are presented as mean ± SEM.

### Data availability

All the relevant data are available from the corresponding author upon reasonable request. Microarray data has been deposited in Array Express database and the accession number is E-MTAB-6024.

## Electronic supplementary material

Supplementary Information
