# Supplementary material for: Irisin is a pro-myogenic factor that induces skeletal muscle hypertrophy and rescues denervation-induced atrophy
Source: Nat Commun. 2017 Oct 24;8:1104. doi: 10.1038/s41467-017-01131-0 (PMC5653663; doi:10.1038/s41467-017-01131-0)
Supplement: Supplementary file 1 — Supplementary Information [file 41467_2017_1131_MOESM1_ESM.pdf]

Supplementary Figure 1: Full western blot scans for Figure 2g

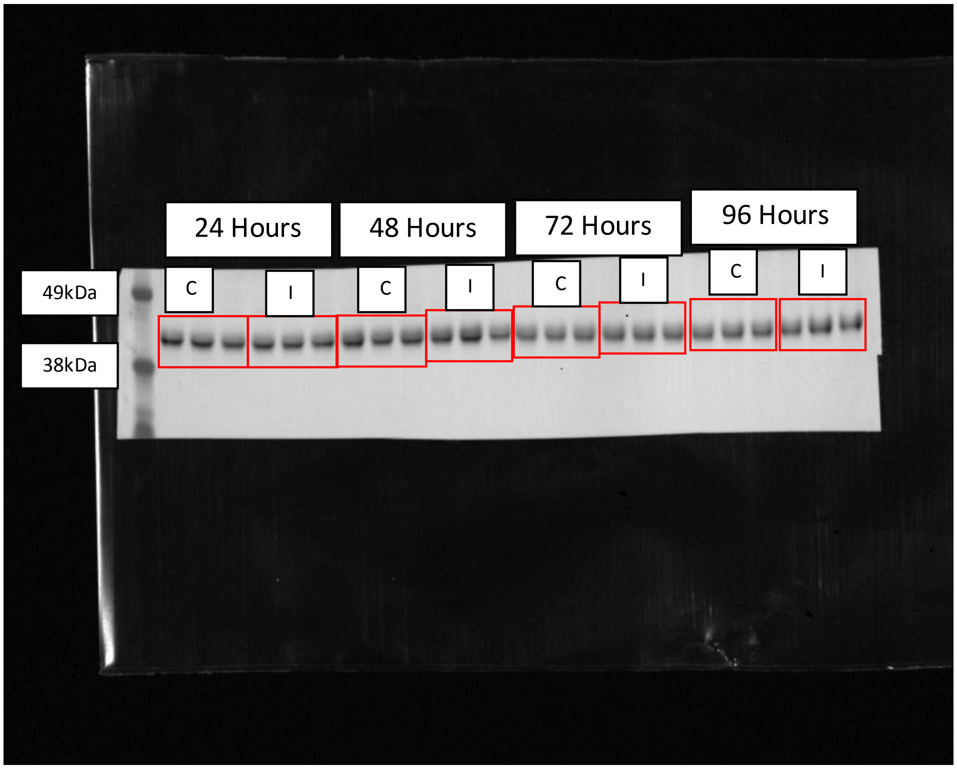

MyoD Western blot scan

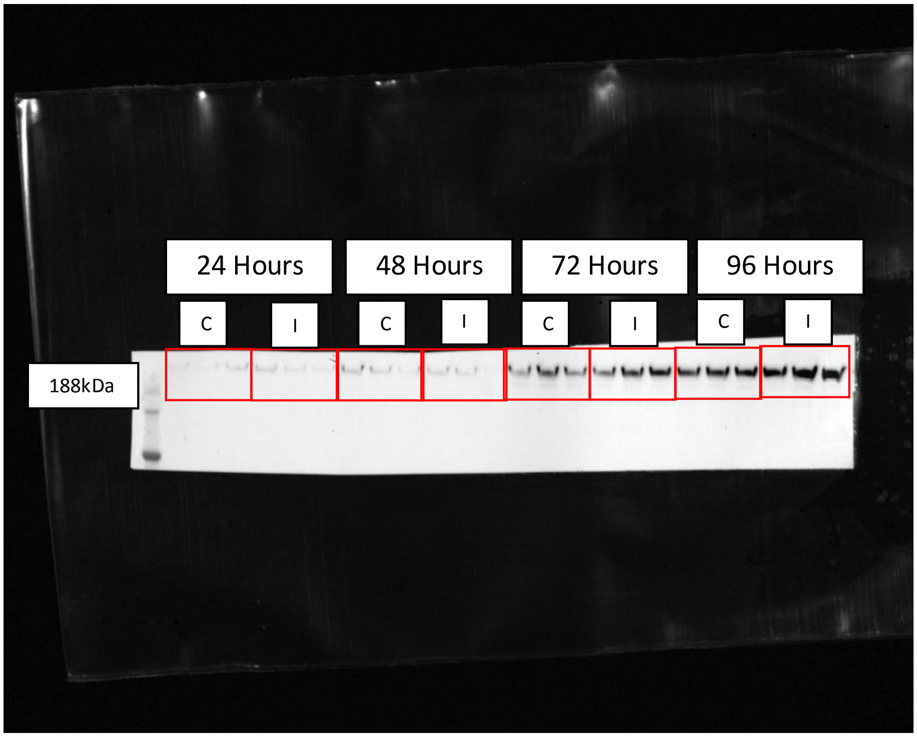

MHC Western blot scan

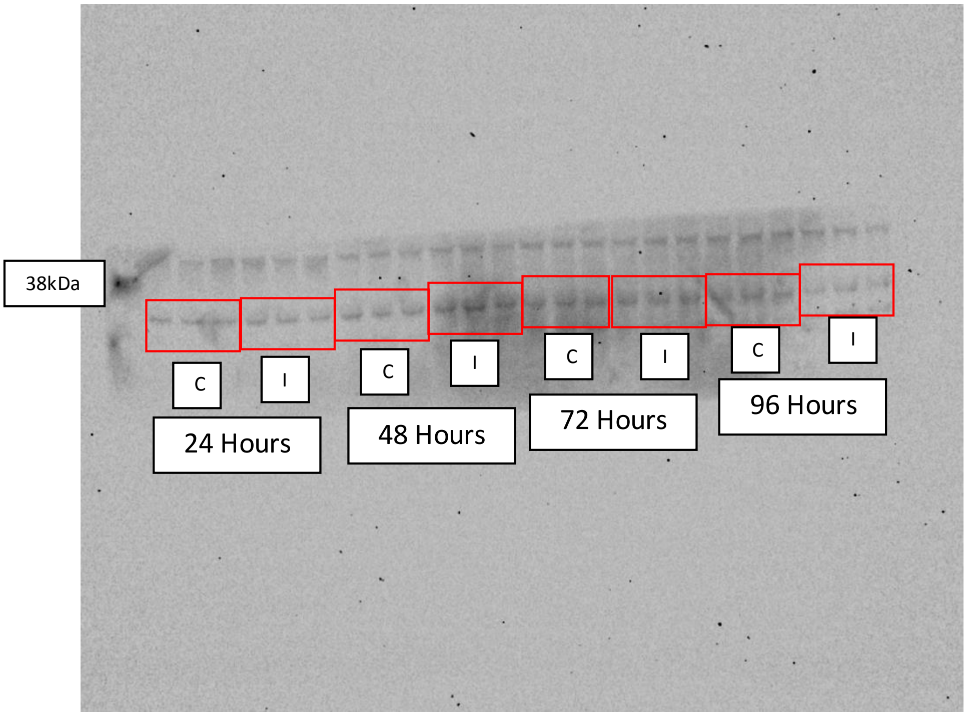

Myogenin Western blot scan

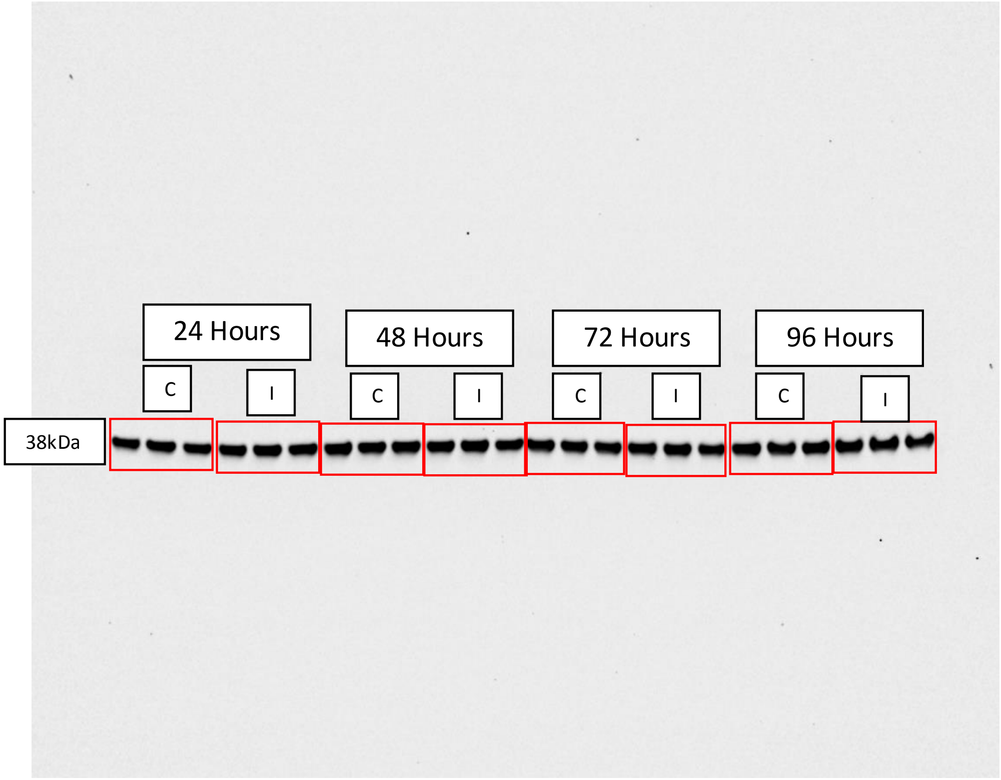

GAPDH Western blot scan for MyoD, MHC and Myogenin

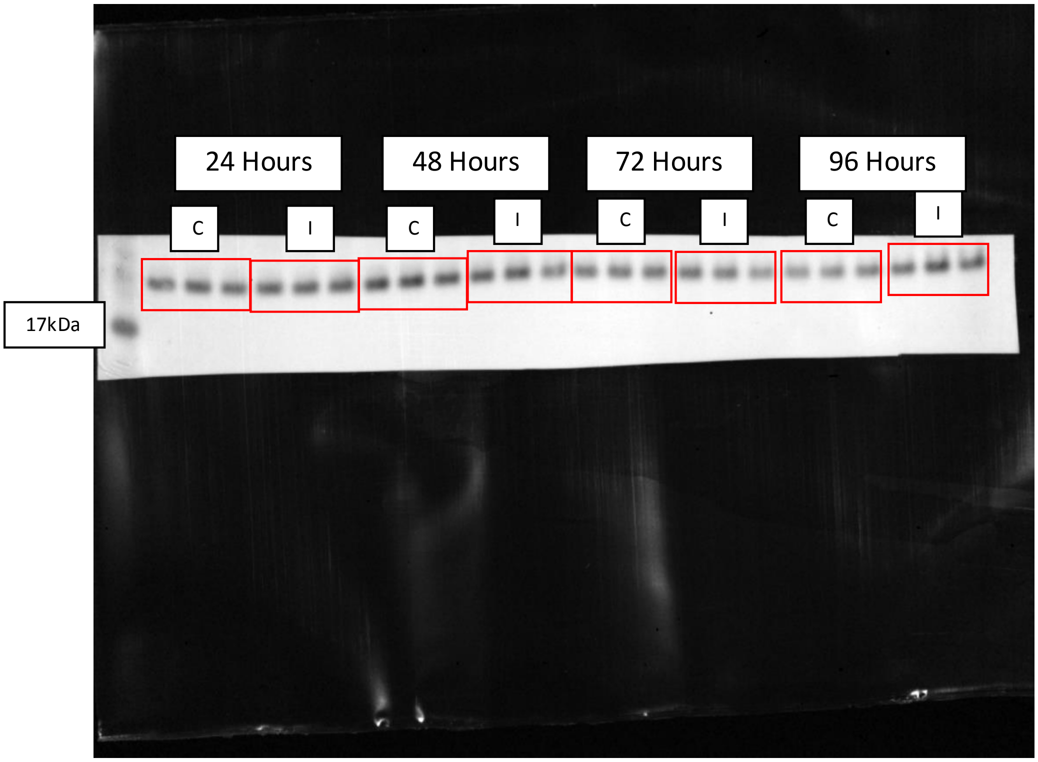

P21 Western blot scan

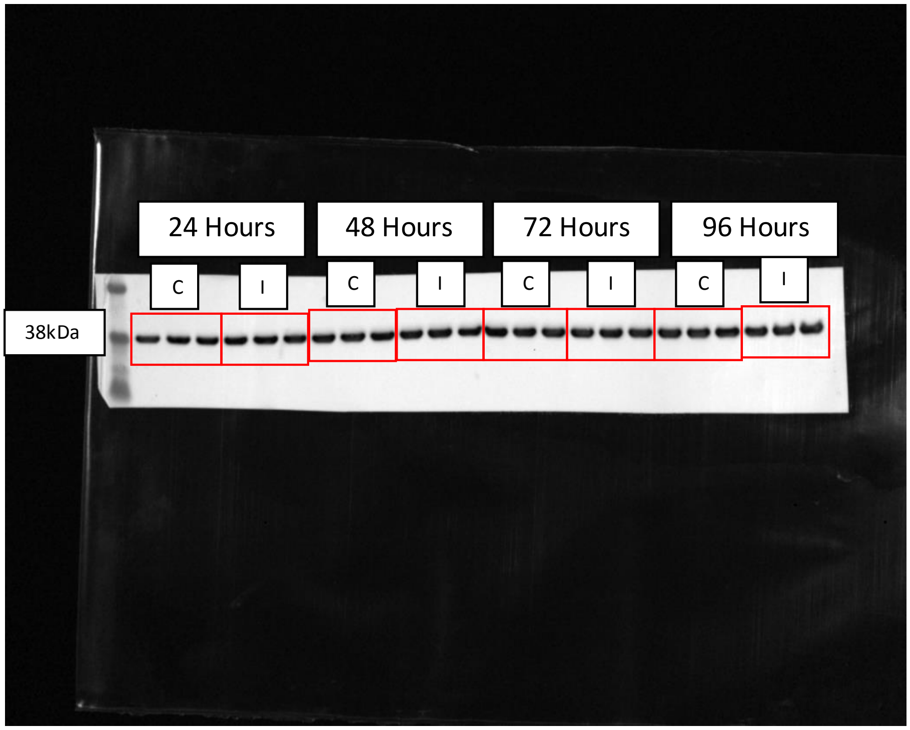

GAPDH Western blot scan for p21

Supplementary Figure 2: Full western blot scans for Figure 4b(i)

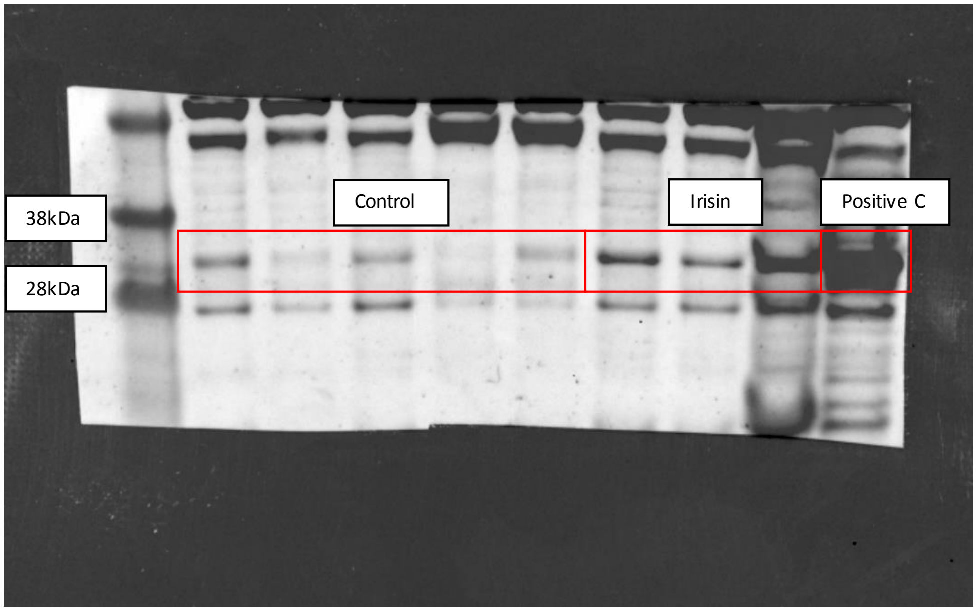

Western blot scan for Ucp1

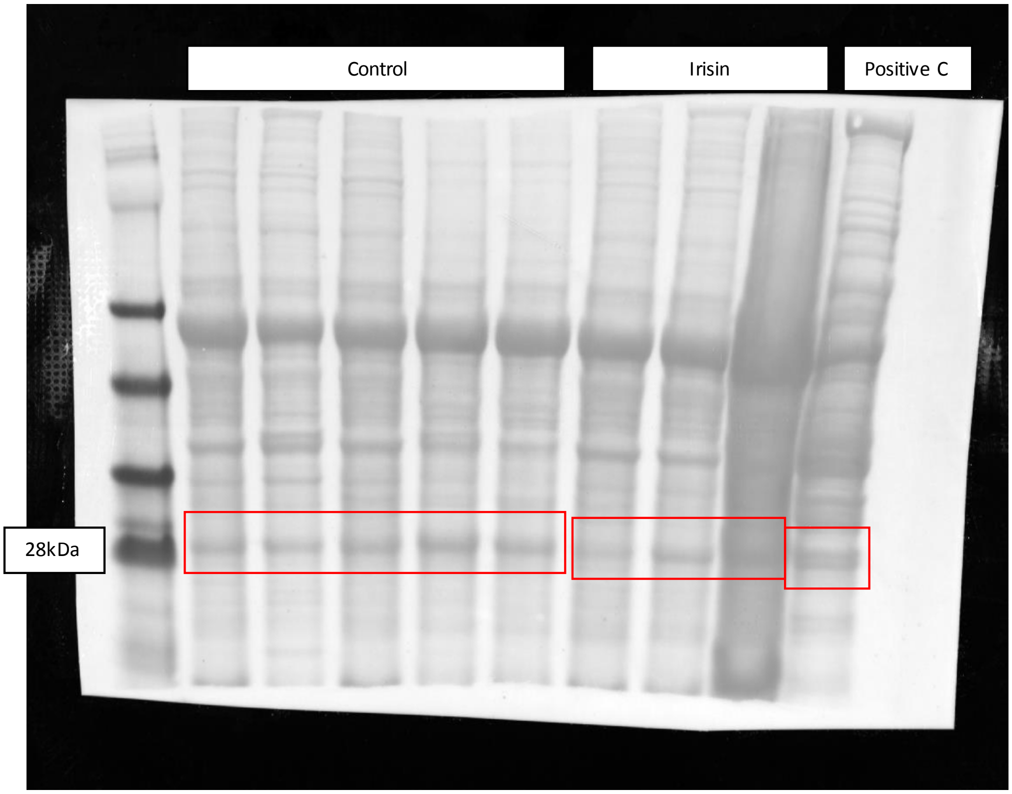

Ponceau Image for Ucp1

Supplementary Figure 3: Full western blot scans for Figure 5a

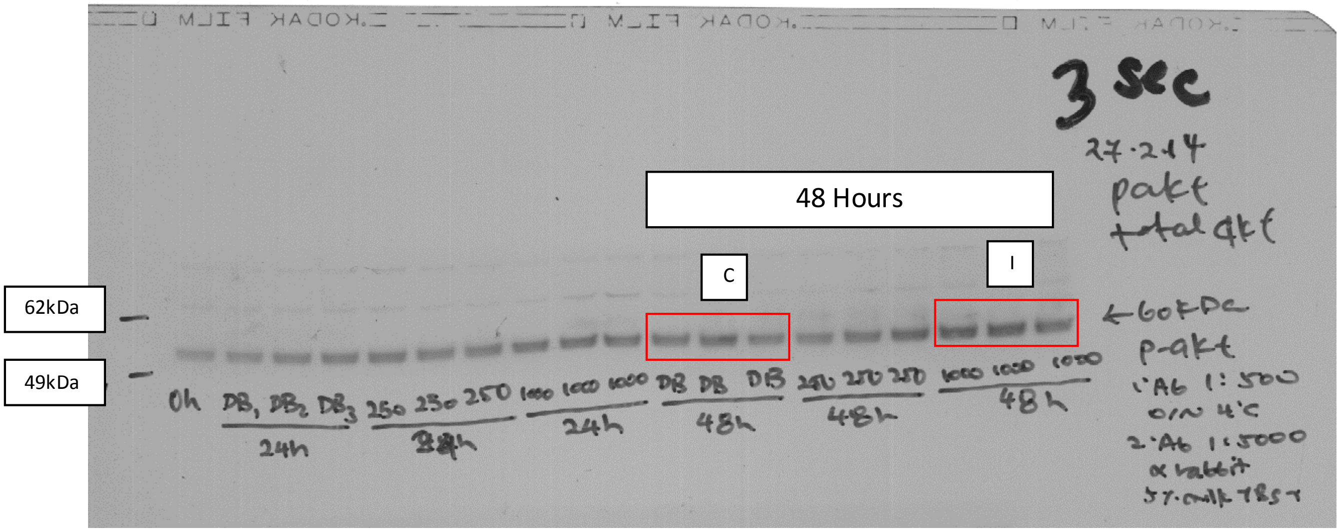

p-Akt Western blot scan- *irrelevant western blot is not shown*

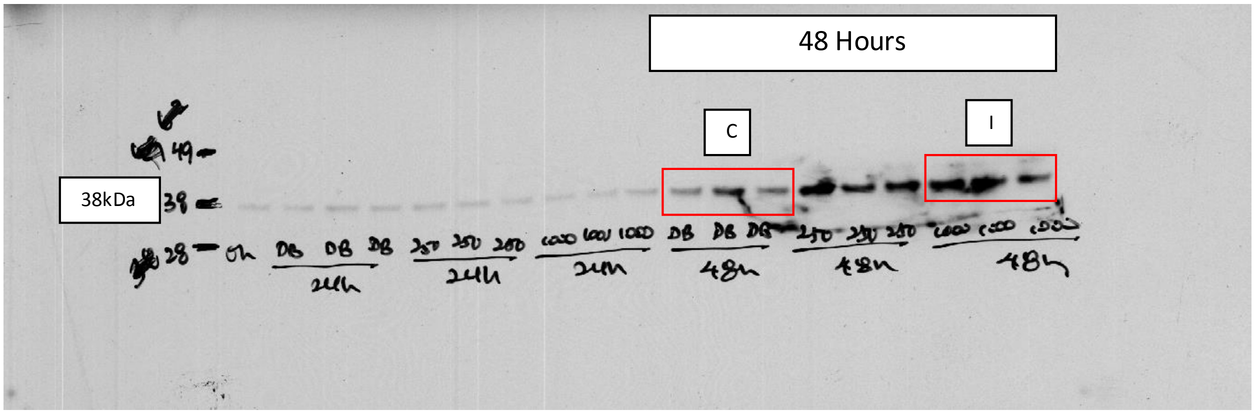

p-Erk Western blot scan- *irrelevant western blot is not shown*

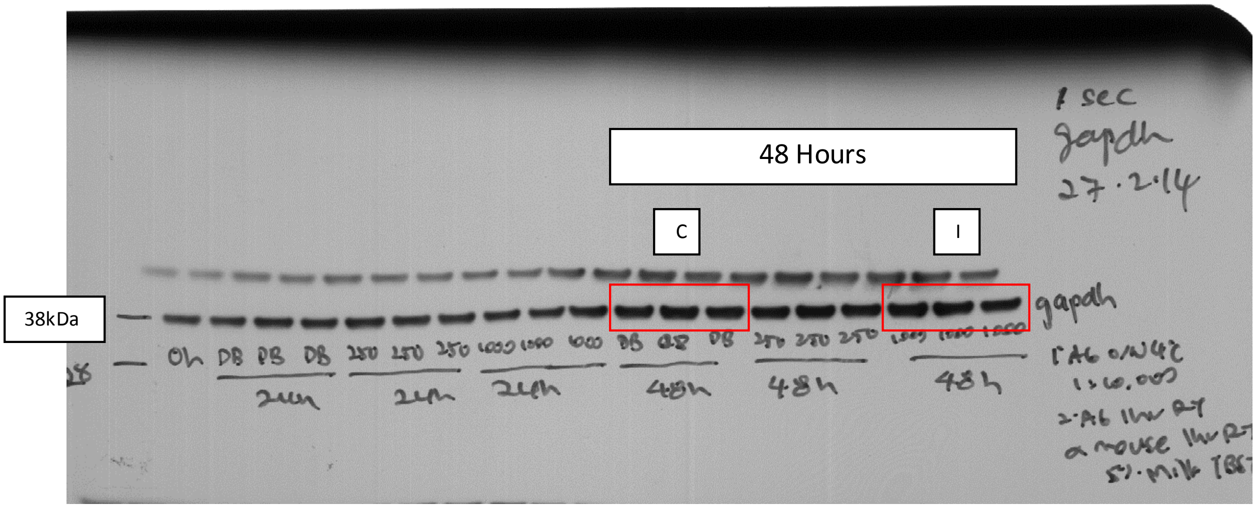

GAPDH Western blot scan for p-Akt and p-Erk

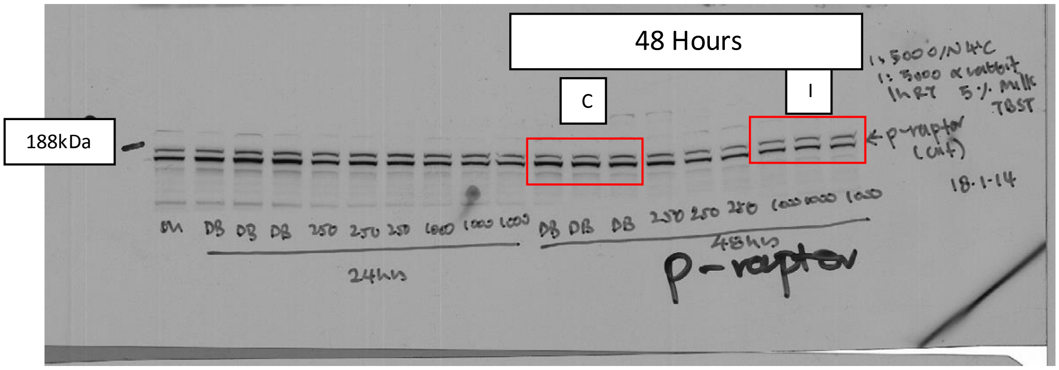

p-raptor Western blot scan - *irrelevant western blot is not shown*

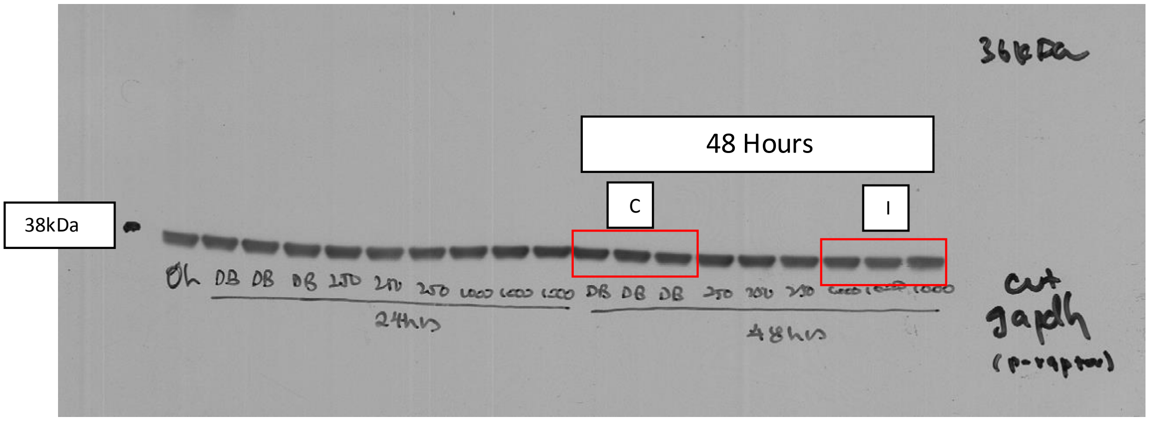

GAPDH Western blot scan for p-raptor- *irrelevant western blot is not shown*

Supplementary Figure 4: Full western blot scans for Figure 5b

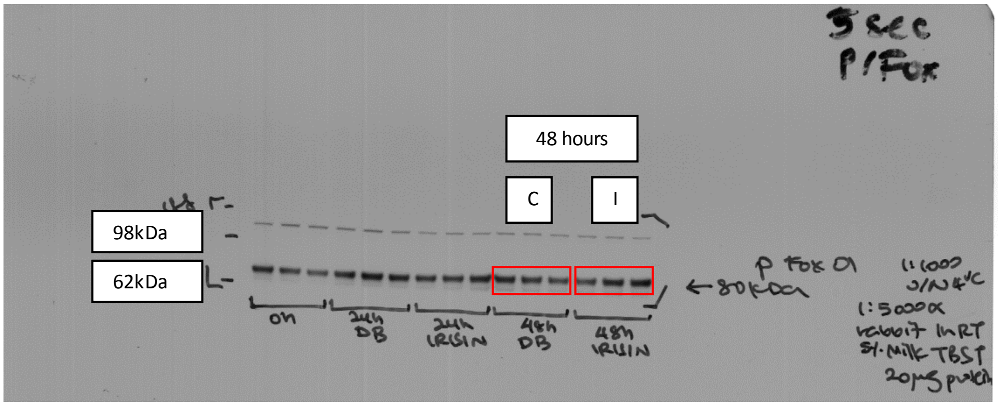

p-FoxO1 Western blot scan- *irrelevant western blot is not shown*

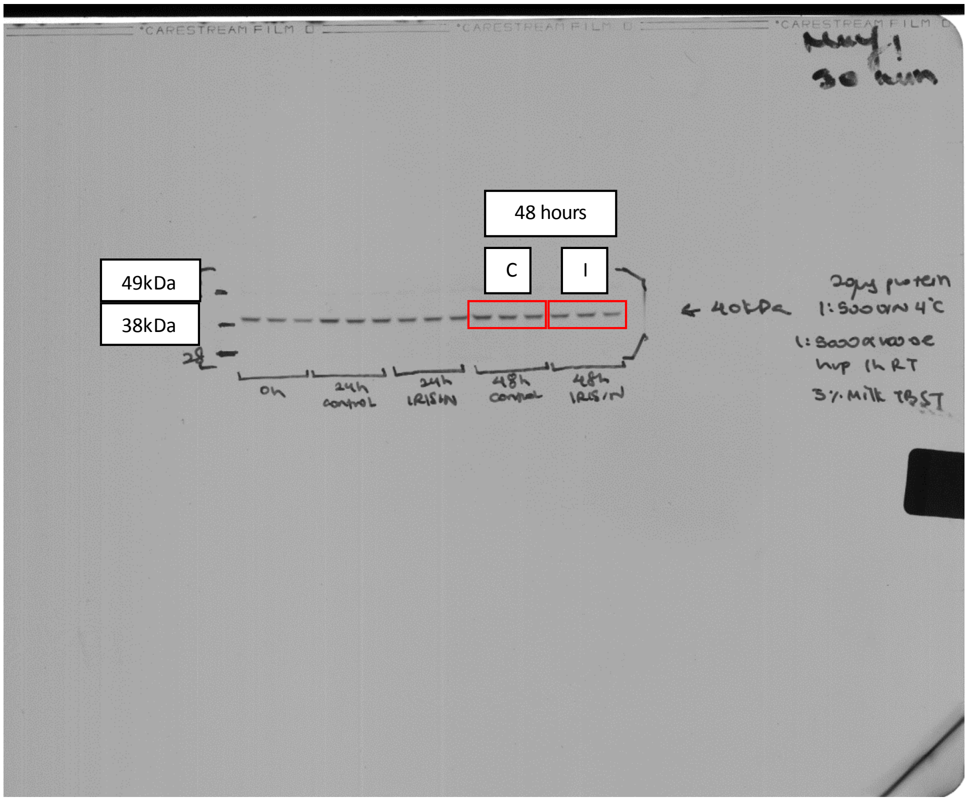

Murf1 Western blot scan

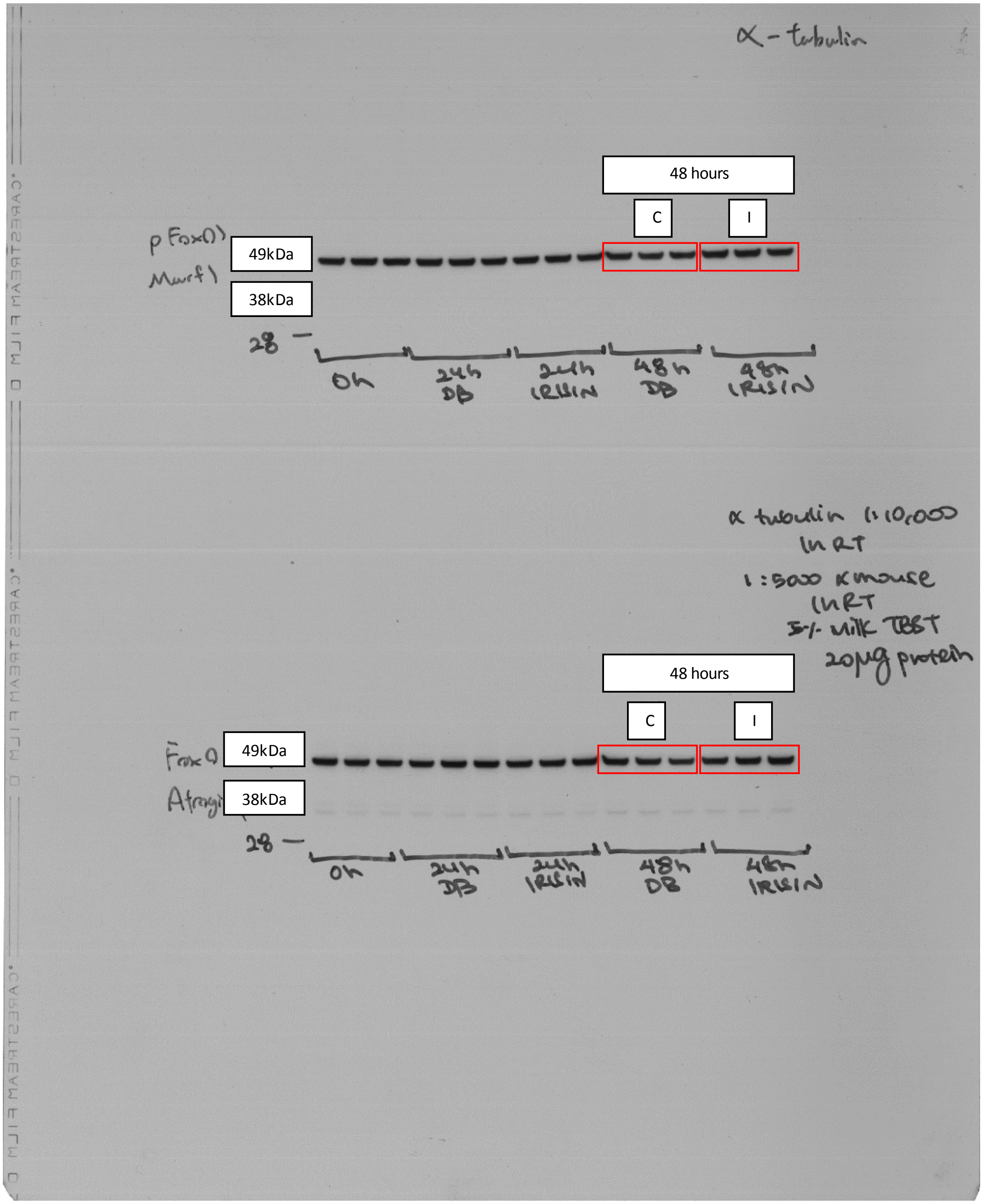

α-tubulin Western blot scan for p-FoxO1 and Murf1- top blot

α-tubulin Western blot scan for FoxO1 bottom blot

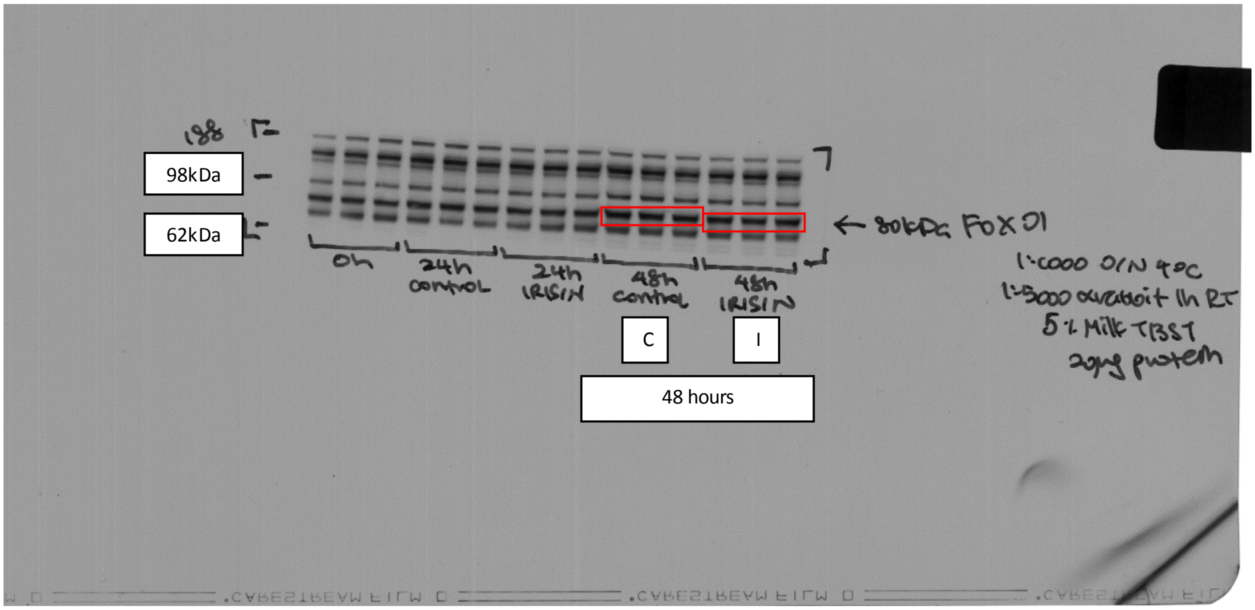

FoxO1 Western blot scan- *irrelevant western blot is not shown*

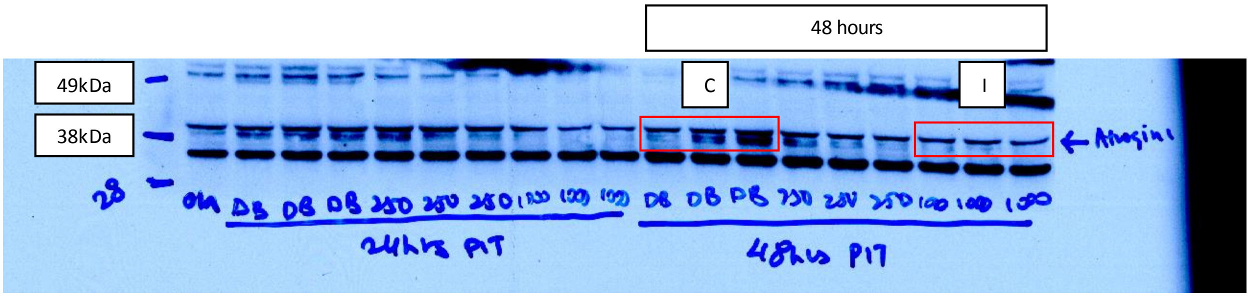

Atrogin-1 Western blot scan

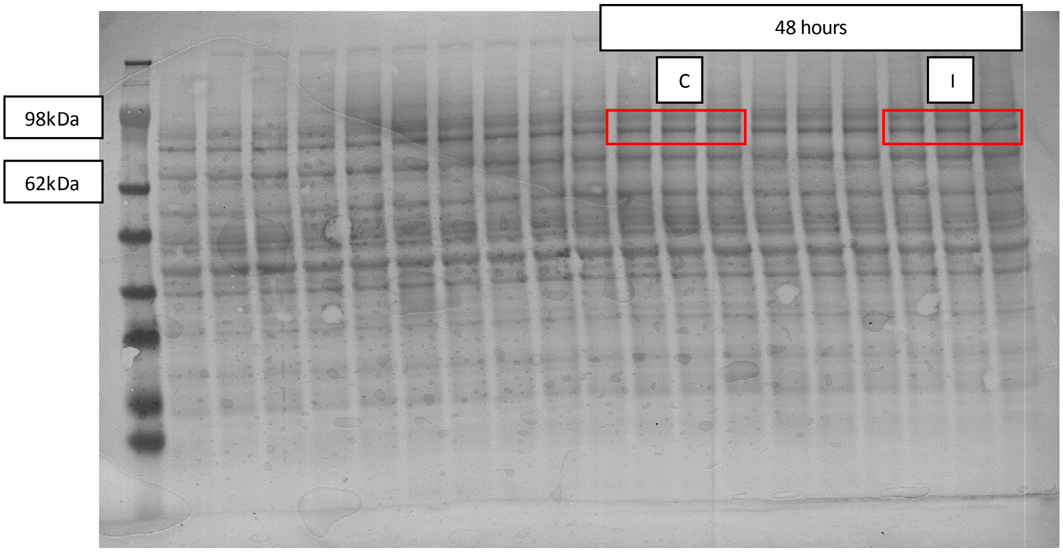

Ponceau S staining for Atrogin-1

Supplementary Figure 5: Full western blot scans for Figure 9c

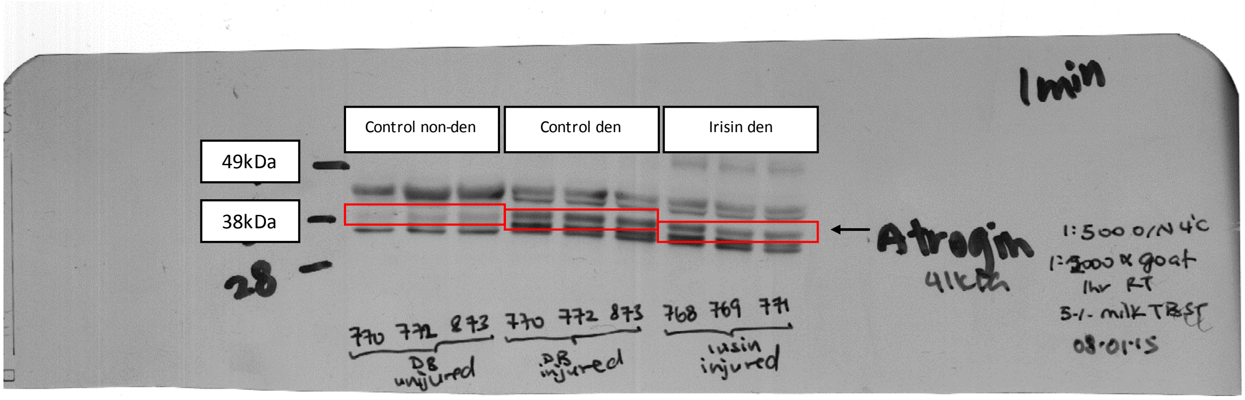

Atrogin-1 Western blot scan

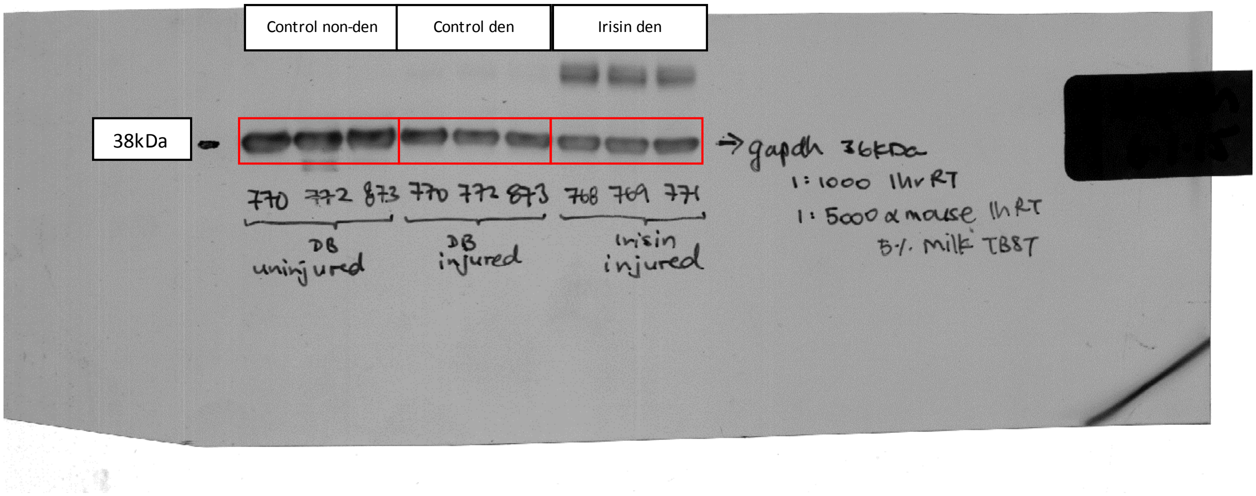

GAPDH Western blot scan for Atrogin-1

Supplementary Figure 6: Full western blot scans for Figure 9e

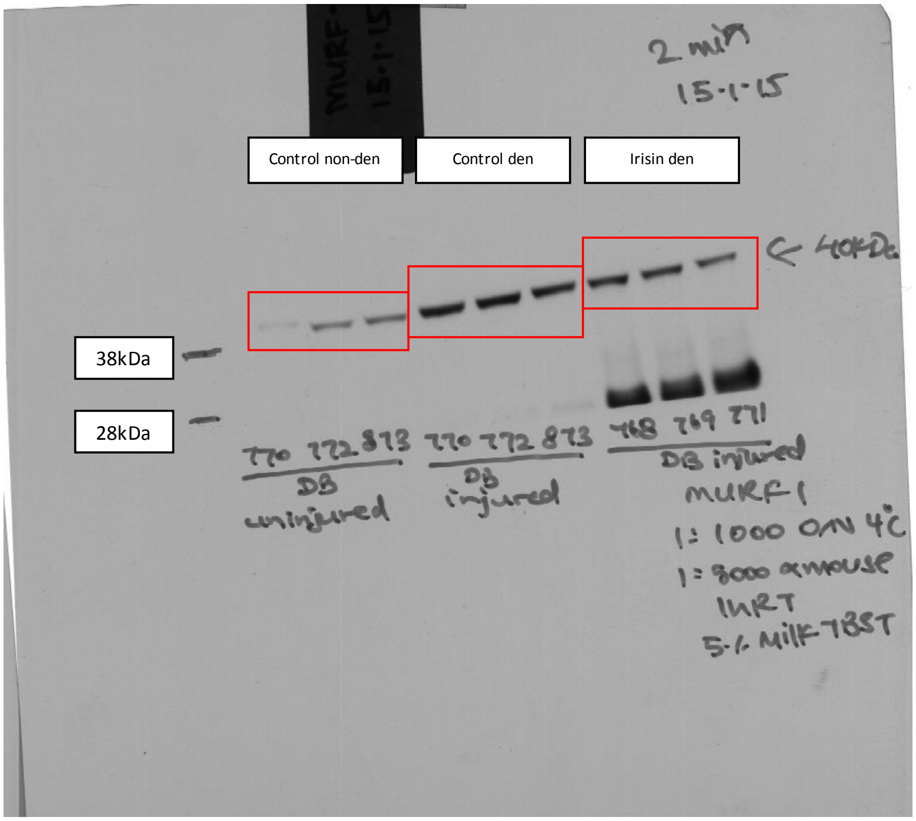

Murf1 Western blot scan

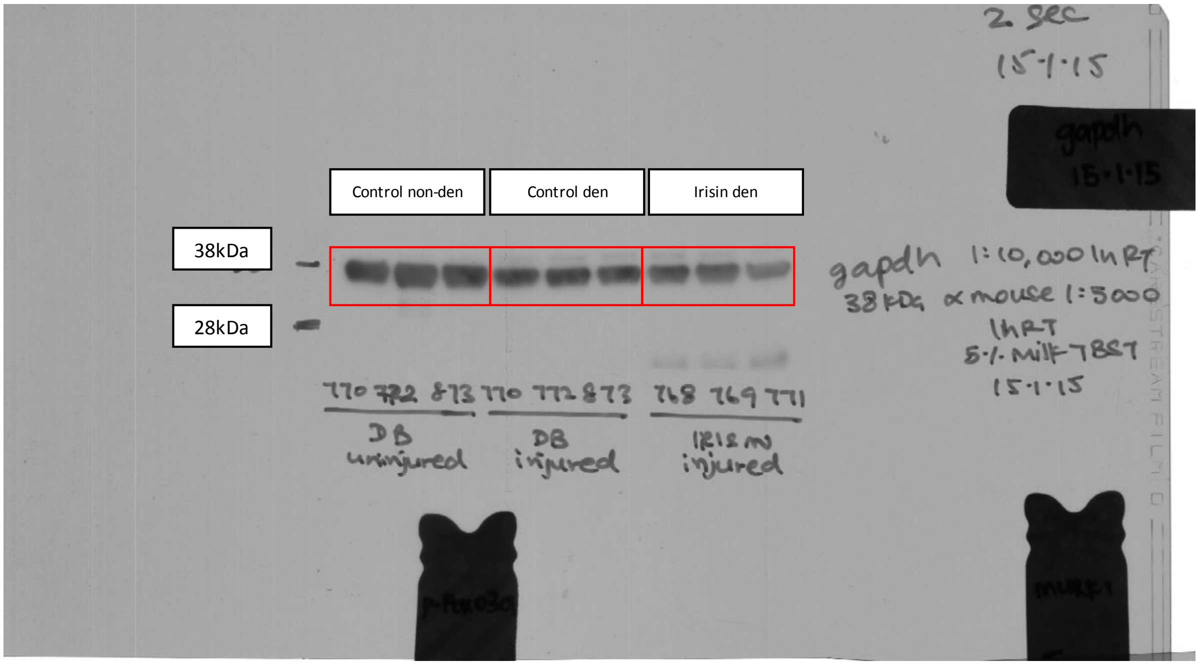

GAPDH Western blot scan for Murf1
